# Propagation of side-stream partial nitritation–anammox disturbance to mainstream wastewater greenhouse gas emissions

**DOI:** 10.1038/s41545-026-00600-8

**Published:** 2026-06-24

**Authors:** Zhufang Wang, Xiaobo Shen, Bang Du, Wenhan Li, Paul Church, Claudio Avignone-Rossa, Kai Yang, Bing Guo

**Affiliations:** 1 School of Engineering, University of Surrey, Guildford, UK; 2 Thames Water Utilities Ltd, Clearwater Court, Reading, UK; 3 Department of Mathematics, University of York, York, UK; 4 Microbes, Infection and Immunity Division, University of Surrey, Guildford, UK; 5 Advanced Technology Institute, University of Surrey, Guildford, UK

**Keywords:** Environmental sciences, Microbiology

## Abstract

Side-stream partial nitritation-anammox (PN/A) process is energy efficient for nitrogen removal but highly sensitive to disturbances such as high organic loadings from anaerobic digestion (AD) liquor. How such disturbance affects downstream treatment performance and greenhouse gas (GHG) emissions in full-scale plants remains insufficiently understood. Here, a full-scale Anoxic-Aerobic-AD-PN/A wastewater treatment plant was compared during satisfied and disturbed PN/A operation. Under disturbed operation, the mainstream apparent liquid-phase TN removal rate decreased from 472.4 to 298.2 kg-N/d and estimated N_2_O and CH_4_ emissions increased by 69.39 kg-N_2_O/day and 10.99 kg-CH_4_/day, respectively. Estimated mainstream GHG emissions rose from 61784.2 to 82768.9 CO_2_-eq.kg/day. A broader comparison including PN/A N_2_O estimates and biogas-electricity offset also suggested increased climate burden (58997.2 to 77742.1 CO_2_-eq kg/day). Metagenomic analysis showed marked reductions in the relative abundance of *Ca.* *Brocadia* and *Nitrosomonas* in PN/A, together with a strong depletion of anammox genes, *hdh*/*hzo*. Elevated *Nitrosomonas*-associated cytochrome P460 and higher abundances of *nirK/norBC* (assigned to *Nitrosomonas* and *Nitrospira*) in the Anoxic and Aerobic tanks were consistent with increased nitrifier-associated N_2_O-production potential. These findings indicate that disturbance of side-stream PN/A can propagate to the mainstream and increase estimated mainstream GHG emissions, highlighting the need to balance AD intensification with stable PN/A operation.

## Introduction

Wastewater treatment plants (WWTPs) play a pivotal role in nutrient removal, yet significant challenges remain due to elevated nitrogen levels in wastewater, leading to eutrophication and degradation of aquatic ecosystems^1,2^. Another challenge is that WWTPs are contributors to greenhouse gas (GHG) emissions, including N₂O and CH₄. Globally, WWTPs rank as the third-largest source of N₂O and fifth-largest source of CH₄^3^, with those gases exhibiting global warming potentials 298 and 28 times higher than CO₂, respectively, over a 100-year period^4,5^. Thus, optimizing WWTPs to simultaneously enhance nitrogen removal and reduce GHG emissions is critical to achieving environmental sustainability.

The partial nitritation-anammox (PN/A) process has emerged as a sustainable alternative to conventional nitrogen removal processes. Unlike energy-intensive nitrification/denitrification, PN/A employs an autotrophic pathway where anammox bacteria (AnAOB) oxidize NH₄^+^ using NO₂^−^ to produce N₂, reducing aeration demand by ~60% under low- or non-COD conditions, eliminating organic carbon requirements, and cutting sludge production by ~80%^6^. By integrating partial nitritation (NH₄^+^ to NO₂^−^) with anammox, PN/A avoids external NO₂^−^ dosing, making it ideal for NH₄^+^-rich, NO₂^−^-scarce wastewater like anaerobic digestion (AD) dewatering liquor^7^. Furthermore, PN/A has been reported to reduce N_2_O emissions by 62%–90% compared to traditional methods^8,9^.

However, the PN/A process faces significant challenges when treating complex wastewater like AD dewatering liquor. The core microorganisms of PN/A, AnAOB and ammonium-oxidizing bacteria (AOB) are highly sensitive to organic substances, with optimal performance typically requiring a low influent C/N ratio of 0.5^10,11^. The complex organic matter in AD dewatering liquor could disrupt PN/A stability, suppressing AnAOB activity and nitrogen removal^12^. For instance, Zhang et al. reported that complex organics directly hindered the stability of the PN/A process^13^. Zhang et al. observed a decline in AnAOB activity from 97% to 72% when treating dewatering liquor where NH_4_^+^ and chemical oxygen demand (COD) (colloidal) increased by 713 mg-N/L and ~700 mg/L, respectively^12^. Similarly, in a PN/A system treating dewatering liquor from high-solid-sludge AD, the nitrogen removal rate decreased from 0.44 to 0.15 kg-N/(m^3^ d) as the proportion of dewatering liquor in influent increased from 73.3% to 100%^14^. These studies highlight the vulnerability of PN/A treating AD dewatering liquor. Previous studies have primarily been conducted in lab-scale or pilot-scale reactors, leaving a gap in understanding the resilience of full-scale PN/A receiving AD dewatering liquor, and its impact on whole-process nitrogen removal. When side-stream PN/A is impaired, the high-nitrogen stream directly enters the mainstream, causing increased nitrogen loading burdens.

In addition to nitrogen removal, GHG emissions present another challenge. N_2_O can be produced during nitrification and denitrification, while CH_4_ may be released through unintended pathways such as leakage, dissolution and stripping, despite AD being designed for full capture and combustion^15,16^. Globally, wastewater treatment contributes 7–10% of anthropogenic CH_4_ emissions^17^. N_2_O emissions in PN/A or nitrification/denitrification systems range from 0.17 to 5.1% of the total influent nitrogen load^18^. For instance, N_2_O emissions from a PN/A bioreactor treating dewatering liquor containing approximately 1000 mg-N/L of NH_4_^+^ ranged from 0.4 to 4.6% of the nitrogen load^19^. It was reported that 1.2% of the total nitrogen in the influent was converted into N_2_O due to heterotrophic denitrification in a pilot-scale PN/A system^20^. Schaubroeck et al. found that the N_2_O emissions from a full-scale side-stream PN/A system were significantly lower than those from the mainstream Anoxic-Oxic system^21^. However, CH_4_ and N_2_O emissions in the mainstream WWTPs lack monitoring data, particularly in the scenario of a disturbed PN/A process, which remains a critical knowledge gap.

To address these gaps, this study evaluated the impact of disturbed side-stream PN/A process on nitrogen removal and GHG emissions in a full-scale WWTP in the UK. The WWTP employs an integrated Anoxic-Aerobic-AD-PN/A configuration, in which indigenous and externally imported sludge are fed to the AD unit for renewable biogas recovery. During the disturbed phase, increased externally imported sludge loading enhanced CH_4_ recovery but coincided with impaired PN/A performance. This study examined (i) nitrogen removal efficiency across the AD, PN/A, Anoxic, and Aerobic units; (ii) mainstream GHG emissions estimated from on-site dissolved CH_4_ and N_2_O monitoring; and (iii) microbial community dynamics and the functional potential using a metagenomics approach. Overall, this study provides full-scale observational evidence that side-stream PN/A disturbance can propagate to the mainstream, increasing downstream nitrogen burden and dissolved-gas-based estimated GHG emissions, while metagenomics provides ecological context for the observed process-level changes.

## Results

### Comparison of water quality between the two phases

**Fig. 1:**
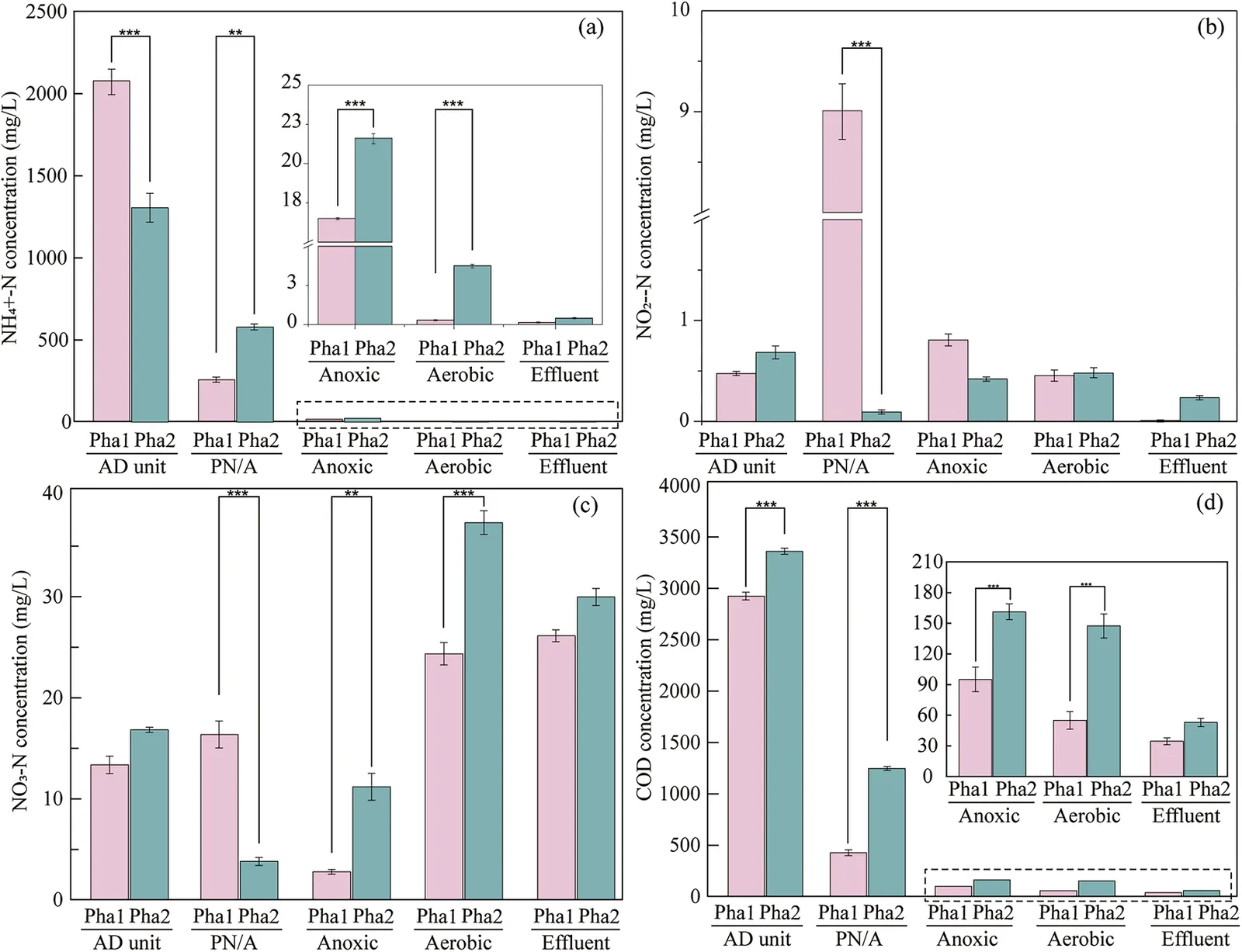
Nitrogen and COD concentrations across treatment units during satisfied and disturbed PN/A operation. **a** NH_4_^+^-N concentrations, **b** NO_2_^−^-N concentrations, **c** NO_3_^−^-N concentrations, **d** COD concentrations in the AD unit, PN/A tank, Anoxic tank, Aerobic tank, and final effluent during Phase 1 and Phase 2. *P*-levels are indicated (*: <0.05, **: <0.01, ***: <0.001), Pha1: Phase 1, Pha2: Phase 2. Statistical comparisons were based on *n* = 9 independent samples per phase.

The nitrogen and COD concentrations in each unit during satisfied PN/A (Phase 1) and disturbed PN/A (Phase 2) phases are presented in Fig. 1. In Phase 1, the AD unit contained 2078.7 mg-N/L NH_4_^+^, 0.48 mg-N/L NO_2_^−^, 13.1 mg-N/L NO_3_^−^, and 2925.7 mg/L COD, corresponding to a C/N ratio of 1.41. In the PN/A tank, NH_4_^+^ and COD decreased to 256.7 mg-N/L and 425.5 mg/L, respectively (*p* < 0.001), giving an NH_4_^+^ removal efficiency of 87.6%. The average ammonium volumetric loading and removal rates were 1.5 and 1.3 kg-N m^-^³ d^-^¹, respectively, indicating stable PN/A performance. NO_2_^−^ and NO_3_^−^ increased to 9.0 mg-N/L and 16.1 mg-N/L, respectively, consistent with partial nitritation converting NH_4_^+^ into NO_2_^−^, which was then used by AnAOB along with NH_4_^+^, producing NO_3_^−^ as a byproduct^22^. However, the observed NO_3_^−^ concentration was lower than expected from a closed stoichiometric anammox balance, suggesting that concurrent NO_3_^−^ reduction/denitrification and full-scale hydrodynamic effects also influenced the PN/A effluent composition. Because readily biodegradable COD (rbCOD) in the AD dewatering liquor was not directly measured, the contribution of heterotrophic denitrification could not be quantitatively resolved.

The PN/A effluent was subsequently returned to the mainstream Anoxic tank, where NO_3_^−^, NO_2_^−^, and COD decreased to 2.7, 0.81, and 95.1 mg/L, respectively. In the Aerobic tank, NH_4_^+^ and COD were further reduced to 0.28 mg-N/L NH_4_^+^ and 55.0 mg/L, while NO_3_^−^ increased to 23.9 mg-N/L, indicating nitrification in the aerobic stage. The final effluent contained 0.14 mg-N/L NH_4_^+^, 0.05 mg-N/L NO_2_^−^, 25.7 mg-N/L NO_3_^−^, 34.6 mg/L COD, and 26.0 mg-N/L TN.

In Phase 2, increased external sludge import to the AD unit significantly increased AD COD to 3361.2 mg/L (*p* < 0.001) and resulted in higher COD concentrations in subsequent tanks (PN/A: 1247.2 mg/L, Anoxic: 161.2 mg/L, and Aerobic: 147.3 mg/L) (Fig. 1d). The decreased NH_4_^+^ in the AD unit was probably due to insufficient sludge digestion under higher sludge import. This resulted in an increased C/N ratio of 2.57. In the PN/A tank, NH_4_^+^ increased to 577.1 mg-N/L, which was 2.25 times higher than in Phase 1, while NH_4_^+^ removal efficiency declined from 87.6% to 55.8%. The NH_4_^+^ removal rate also decreased sharply from 1.3 to 0.5 kg-N m^-^³ d^-^¹, despite a lower ammonium volumetric loading of approximately 1.0 kg-N m^-^³ d^-^¹. Meanwhile, NO_2_^−^ and NO_3_^−^ decreased to 0.10 and 3.7 mg-N/L, respectively, indicating inhibited PN/A activity.

The disturbed PN/A effluent subsequently increased the NH_4_^+^, NO_3_^−^, and COD burden in the mainstream tanks. In the Anoxic and Aerobic tanks, NH_4_^+^ increased to 21.6 and 3.8 mg-N/L, respectively, while NO_3_^−^ increased to 11.0 and 36.7 mg-N/L in Phase 2. The higher NO_3_^−^ concentrations likely reflected increased NH_4_^+^ transfer to the mainstream and enhanced downstream nitrification. COD also remained higher in the Anoxic and Aerobic tanks than in Phase 1. Although COD decreased markedly from the PN/A tank to the Anoxic tank, with a larger absolute reduction in Phase 2 (821.7 mg/L) than in Phase 1 (330.4 mg/L), the residual COD remained elevated in Phase 2, suggesting increased side-stream organic loading and incomplete removal within the available downstream treatment capacity.

Consequently, the final effluent in Phase 2 contained NH_4_^+^: 0.42 mg-N/L, NO_2_^−^: 0.23 mg-N/L, NO_3_^−^: 29.5 mg-N/L, COD: 53.0 mg/L, and TN: 30.6 mg/L. Based on the liquid-phase TN load entering and leaving the mainstream process, although the mainstream TN loading was lower in Phase 2 because of reduced influent flow, the TN removal rate decreased from 472.4 to 298.2 kg-N/d, and the TN removal efficiency decreased from 30.6% to 22.4%. The relatively modest TN removal efficiency was mainly associated with NO_3_^−^ accumulation in the final effluent, which was not subject to a site-specific discharge limit. Although regulated effluent parameters remained within the applicable UK discharging limits during both phases^23^, effluent NH_4_^+^, NO_2_^−^, NO_3_^−^, and COD were 2.93-, 4.60-, 1.15-, and 1.53-fold higher than in Phase 1, respectively.

To further compare PN/A sludge activity between the two phases, particularly anammox activity, the functional capacity of the PN/A biomass was evaluated. Because online pathway-specific activity monitoring was not available for the side-stream PN/A tank, off-line specific anammox, nitrification, and denitrification activity tests were conducted using fresh sludge collected from the PN/A tank for both phases. In Phase 1, the specific anammox activity reached 428.4 mg-N g-VSS^-1^ d^-1^, while nitrification and denitrification activities were 172.1 mg-N g-VSS^−1^ d^−1^ (NH_4_^+^ removal), and 106.2 mg-N g-VSS^−1^ d^−1^ (NO_2_^−^/NO_3_^−^ removal) (Supplementary Information, Fig. S1a–c, g). Anammox was therefore the dominant nitrogen removal pathway, exhibiting a rate 4.03 times higher than denitrification. In Phase 2, all three activities declined sharply: anammox to 68.7 mg-N g-VSS^−1^ d^−1^, nitrification to 46.0 mg-N g-VSS^−1^ d^−1^, and denitrification activities to 92.7 mg-N g-VSS^−1^ d^−1^ (Fig. S1d–f, g).

Overall, the disturbed PN/A process disrupted the function of downstream tanks, resulting in higher effluent NH_4_^+^ (+0.27 mg-N/L), NO_2_^−^ (+0.18 mg-N/L), NO_3_^−^ (+3.8 mg-N/L), COD (+18.4 mg/L), TN (+4.6 mg-N/L).

### Greenhouse gas emissions

**Fig. 2:**
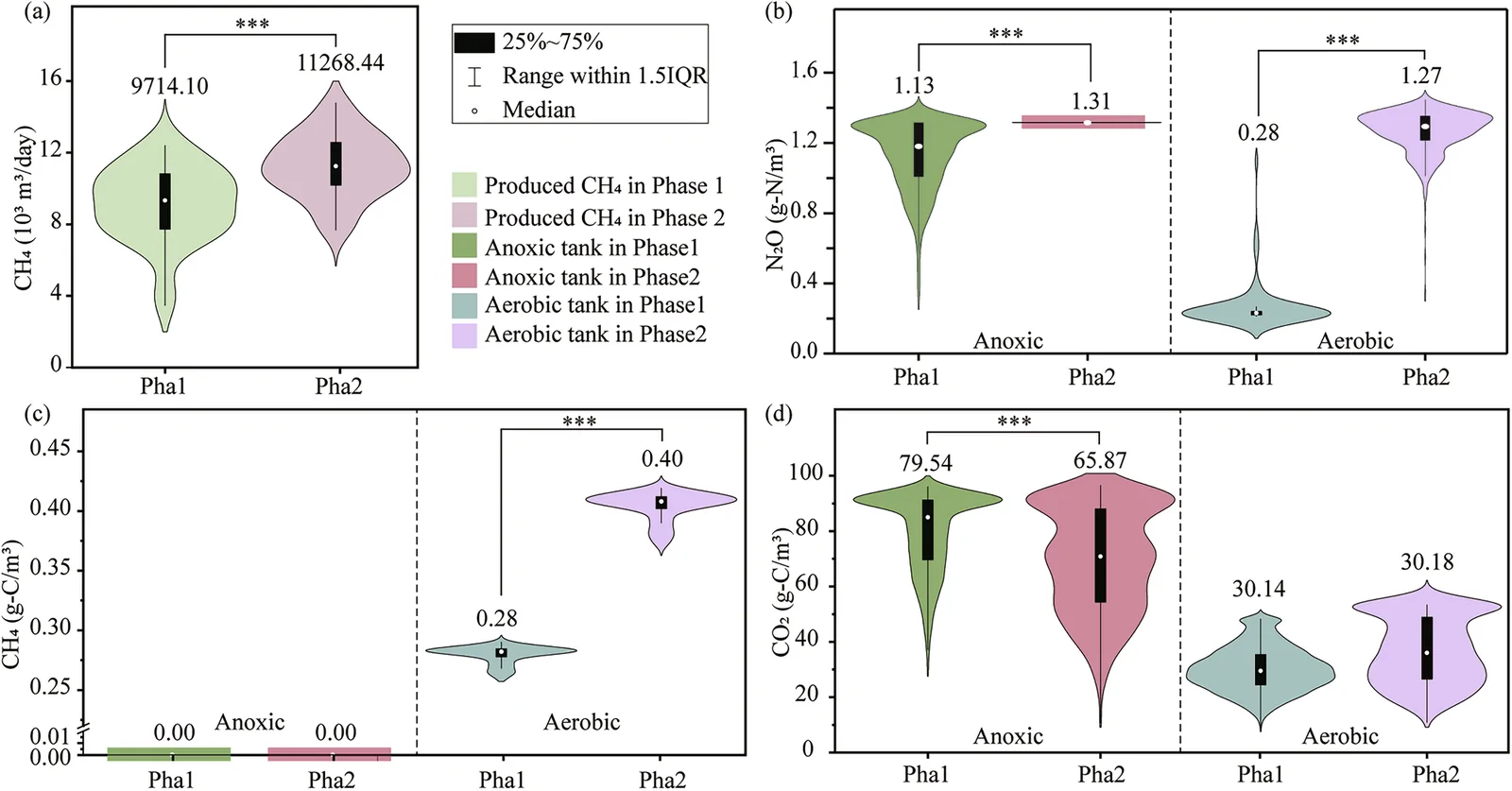
Gas data collected onsite. **a** Produced CH_4_ from the AD tank. Dissolved N_2_O (**b**), CH_4_ (**c**), and CO_2_ (**d**) concentrations. Statistical comparisons were based on *n* = 31 (**a**) and *n* = 778 (**b**–**d**) independent samples per phase, respectively.

To examine the impact of disturbed PN/A process on GHG emissions, dissolved N_2_O, CH_4_, and CO_2_ in the Anoxic and Aerobic tanks, and pure CH_4_ produced from the AD unit were collected (Fig. 2). Based on UK Government GHG Conversion Factors (2025)^24^, the biogas-derived electricity offset 1517.4 CO_2_-eq kg/day by increased CH_4_ production from AD unit in Phase2 (Fig. 2a and Table S1). Meanwhile, the AD unit effluent flow to the PN/A tank increased slightly from 650.0 m^3^/day in Phase 1 to 668.1 m^3^/day in Phase 2.

In Phase 1, dissolved N_2_O was 1.13 g-N/m^3^ and 0.28 g-N/m^3^ in the Anoxic and Aerobic tanks, respectively (Fig. 2b). Dissolved N_2_O data were unavailable in PN/A, and the N_2_O emission rate was estimated based on the IPCC guidelines according to Eqs. (8–10). CH_4_ was 0.28 g-N/m^3^ in the Aerobic tank and was not detected in the Anoxic tank. According to Eqs. (1–4), N_2_O emission rates were 174.16 and 27.48 kg/day from the Anoxic and Aerobic tanks, respectively (Table 1). According to Eqs.(5–7), CH_4_ emission rates were 60.52 kg/day from the Aerobic tank. According to Eqs. (8–10), N_2_O emission rates were 6.23 kg/day in the PN/A tank (Tables 1 and S2).

**Table 1:** Estimated greenhouse gas emissions based on dissolved-gas monitoring in mainstream tanks, with factor-based side-stream PN/A N_2_O estimates

	Aerobic tank				Anoxic tank				PN/A tank			
			CO_2-_eq kg/day				CO_2-_eq kg/day				CO_2-_eq kg/day	
	Pha1	Pha2	Pha1	Pha2	Pha1	Pha2	Pha1	Pha2	Pha1	Pha2	Pha1	Pha2
${{\rm{C}}}_{{\mathrm{CH}}_{4}-{\rm{C}}}$(g/m^3^)	0.28	0.40	1694.6	2002.4	N.D.	N.D.	N.D.	N.D.	N.A.	N.A.	N.A.	N.A.
${{\rm{E}}}_{{\mathrm{CH}}_{4}}\,$(kg/day)	60.52	71.52										
${{\rm{C}}}_{{{\rm{N}}}_{2}{\rm{O}}-{\rm{N}}}$(g/m^3^)	0.28	1.27	8190.4	30,728.4	1.13	1.31	51,899.2	50,038.1	9.59	5.86	1857.8	1135.5
${{\rm{E}}}_{{{\rm{N}}}_{2}{\rm{O}}}\,$(kg/day)	27.48	103.12			174.16	167.91			6.23	3.81		
Total eq CO_2_			9885.0	32,730.8			51,899.2	50,038.1			1857.8	1135.5
Total mainstream eq CO_2_ (CO_2-_eq kg/day)				Pha1	61,784.2							
				Pha2	82,768.9							
Expanded plant-level net eq CO_2_ comparison (including estimated PN/A N_2_O and biogas-electricity offset) (CO_2-_eq kg/day)				Pha1	58,997.2							
				Pha2	77,742.1							

N.D. represents Not detected. N.A. represents Not available. Pha1 refers to Phase1, during which the PN/A process was satisfactory; Pha2 refers to Phase2, during which the PN/A process was disturbed.

In Phase 2, estimated PN/A tank N_2_O emissions decreased to 3.81 kg/day (Eqs. 8–10) (Table S2) because PN/A TN removal decreased from 1176.8 to 495.7 kg-N d^−1^. However, dissolved N_2_O concentration rose significantly in downstream tanks: from 1.13 to 1.31 g-N/m^3^ in the Anoxic tank and from 0.28 to 1.27 g-N/m^3^ in the Aerobic tank (*p* < 0.001; Fig. 2b). Due to reduced influent flow in Phase 2 (Fig. S2a), estimated N_2_O emission rates using Eqs. (1–4) reduced from 174.16 to 167.91 kg-N_2_O/day in the Anoxic tank but increased sharply from 27.48 to 103.12 kg-N_2_O/day in the Aerobic tank between phases (Table 1). Overall, N_2_O emission increased by 69.39 kg-N_2_O/day in mainstream, while combined mainstream and side-stream emissions rose by 66.96 kg-N_2_O/day in Phase 2. Dissolved CH_4_ concentration in the Aerobic tank rose from 0.28 to 0.40 g-C/m^3^ (*p* < 0.001) (Fig. 2c), raising estimated CH_4_ emission from 60.52 to 71.52 kg-CH_4_/day (1.18-fold) (Eqs. 5–7). No CH_4_ was detected in the Anoxic tank.

According to GWP metrics (Eq.11), total CO_2_-eq emissions increased from 61784.2 CO_2_-eq kg/day (Phase 1) to 82768.9 CO_2_-eq kg/day (Phase 2), a 1.34-fold increase (20984.7 CO_2_-eq kg/day rise) in mainstream. When factor-based side-stream PN/A N_2_O estimates and biogas-electricity offset were additionally considered, the expanded plant-level comparison still suggested a net increase of 18744.9 CO_2_-eq kg/day in Phase 2.

### Microbial community shifts

**Fig. 3:**
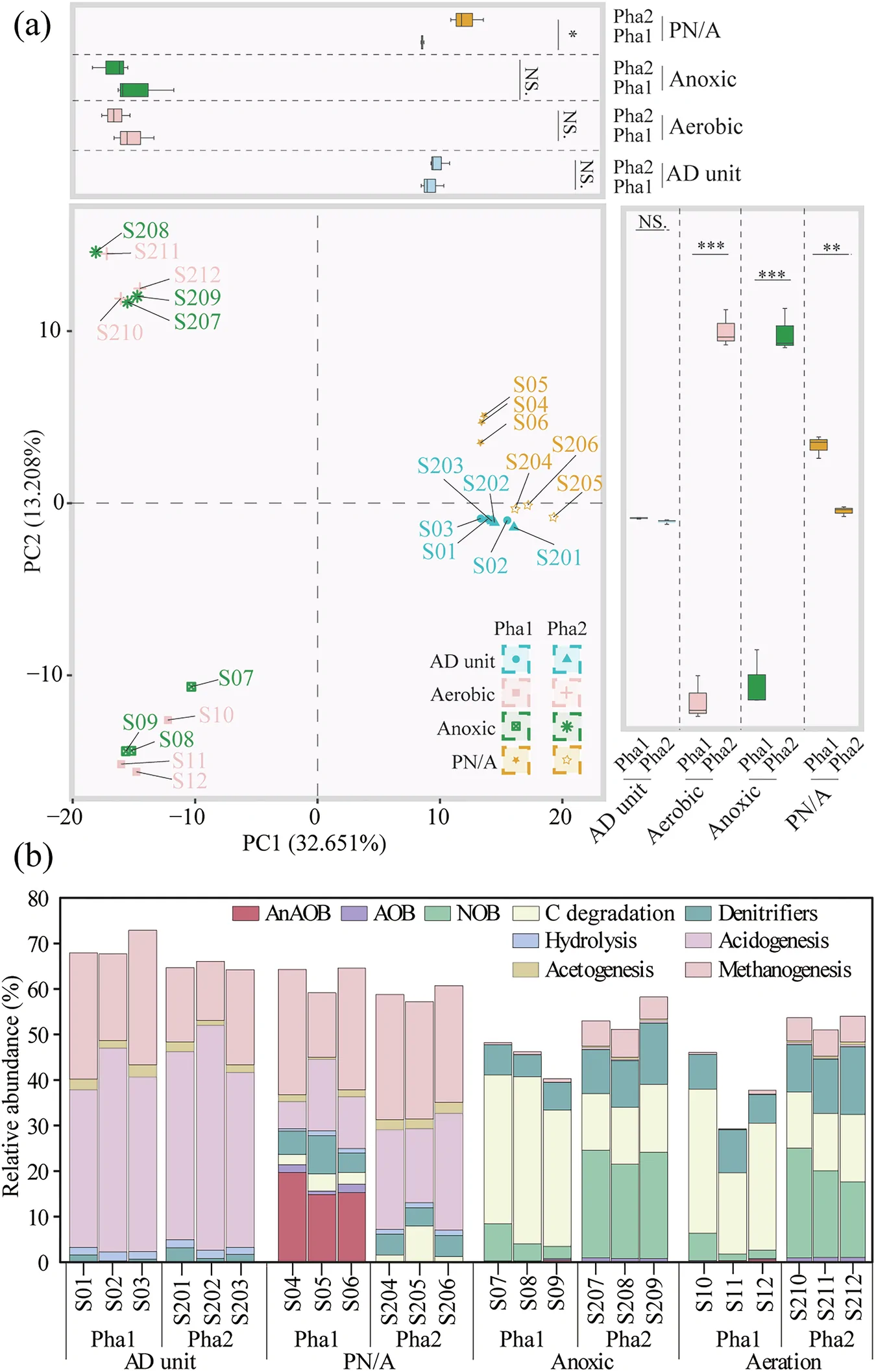
Microbial community dynamics. **a** PCA analysis. Side panels indicate the statistical significance of differences between two phases within each tank (*p < 0.05; **p < 0.01; ***p < 0.001; N.S. stands for Not significant). Statistical comparisons were based on *n* = 3 independent samples per phase. **b** Relative abundance of functional organisms at the genus level.

Alpha diversity indices, including Chao1, Pielou, Shannon, and Simpson, were calculated to characterize microbial community structure across the two phases. Overall, alpha diversity showed phase-dependent changes across treatment units, with detailed results provided in the Supplementary Information (Table S3)^25^. Beta diversity of microbial community structure was analyzed using principal component analysis (PCA) (Fig. 3a). No significant difference was observed in the AD tank between the two phases (*p* > 0.05). However, the PN/A tank community shifted significantly in Phase 2 and clustered closer to the AD unit (*p* < 0.05). This shift reflects the disturbance of the PN/A process and the subsequent dominance by microorganisms from the AD unit, which may outcompete AnAOB under elevated COD conditions^26,27^. The Anoxic and Aerobic tanks exhibited similar microbial community structures, consistent with findings from a full-scale Anaerobic-Anoxic-Aerobic WWTP system^28^. However, the distinct separation between Phase 1 and Phase 2 in the PCA plot indicates significant shifts in the microbial communities of the Anoxic and Aerobic tanks (*p* < 0.05). These shifts could be driven by the side-stream input, which introduced higher NH_4_^+^ and organic carbon levels into the downstream tanks, as well as distinct biomass.

Taxonomy profiling at kingdom and phylum levels showed increased archaeal relative abundance in the Anoxic and Aerobic tanks and a marked decline in Planctomycetes in the PN/A tank in phase 2 (Fig. S3). At the genus level, the AD unit was dominated by bacteria in hydrolysis (*Coprothermobacter*), acidogenesis (*GGB26744* within Firmicutes), and acetogenesis (*Petrimonas*), and methanogenic archaea (*Methanosarcina*) (Fig. 3b and Supplementary Data 1). From Phase 1 to Phase 2, hydrolytic, acidogenic, and acetogenic bacteria increased from 43.2 ± 4.9% to 46.4 ± 5.4%, whereas methanogens decreased from 25.4 ± 5.6% to 16.7 ± 4.0%. In the PN/A tank, AOB (*Nitrosomonas*) declined from 1.45 ± 0.56% to 0.01 ± 0.00%, and AnAOB (*Candidatus Brocadia*) declined from 16.6 ± 2.7% in Phase 1 to undetectable levels in Phase 2 (*p* < 0.05). In contrast, hydrolytic, acidogenic, and acetogenic bacteria increased from 13.0 ± 4.6% in Phase 1 to 24.6 ± 4.9% in Phase 2, and methanogens increased from 22.9 ± 7.4% to 26.3 ± 1.0% (*p* < 0.05). In the Anoxic and Aerobic tanks, AOB (*Nitrosomonas*) increased by 4.4- and 5.4-fold in Phase 2, respectively. NOB (*Nitrospira*) increased from 4.8 ± 2.9% and 3.1 ± 1.5% in Phase 1 to 22.6 ± 1.6% and 19.9 ± 3.8% in Phase 2, respectively. Denitrifiers (*GGB44536* in family Chromobacteriaceae) increased 1.9-fold and 1.6-fold, and *Methanosarcina* rose from 0.41 ± 0.13% and 0.33 ± 0.27% in Phase 1 to 4.9 ± 0.60% and 4.9 ± 0.46% in Phase 2, respectively (*p* < 0.05).

### Microbial functional potential

Broad KEGG-based functional profiling indicated a general shift in metabolic potential during Phase 2, including reduced nitrogen-metabolism potential in the PN/A tank and increased methane-metabolism signatures in downstream tanks (Figs. S4 and S5). Metagenomic profiles of nitrogen-cycling genes were analyzed to evaluate changes in functional potential across the two phases.

In the PN/A tank, the relative abundance of genes encoding NH_4_^+^, NO_3_^−^, and NO_2_^−^ transporters (*amtB*, *narK*, and *nirC*) decreased by 53.7%, 53.6%, and 71.2%, respectively, from Phase 1 to Phase 2 (Fig. S6 and Supplementary Data 2). Genes involved in partial nitrification and anammox also decreased markedly. The abundance of *amoA/B/C* reduced from 71.2 RPKM in Phase 1 to 0.64 RPKM in Phase 2. Likewise, anammox genes *hzs, hdh*, and *hzo* declined from 33.6, 29.5, and 15.7 RPKM in Phase 1 to very low or even undetectable levels in Phase 2.

In the Anoxic and Aerobic tanks, the gene-level response differed from that in the PN/A tank. The relative abundances of *amtB* and *nirC* increased by 4.4–76.1%, whereas *narK* decreased by 28.3% and 16.8%, respectively, suggesting a shift in nitrogen-transport potential under Phase 2 conditions (Fig. S6 and Supplementary Data 2). Nitrification-related genes increased significantly (*p* < 0.05): *amoA/B/C* increased from 45.4 and 41.6 RPKM in Phase 1 to 109.0 and 117.2 RPKM in Phase 2, and *hao* increased by 2.1- and 2.3-fold in Phase 2 in the Anoxic and Aerobic tanks, respectively. For denitrification, *narGHI* and *napAB* increased by 12.6% and 24.4%, *nirK/S* increased slightly (4.7% and 4.9%), *norB/C* increased by 29.6% and 46.5%, and *nosZ* reduced by 0.7% and 9.5% in the Anoxic and Aerobic tanks in Phase 2, respectively.

**Fig. 4:**
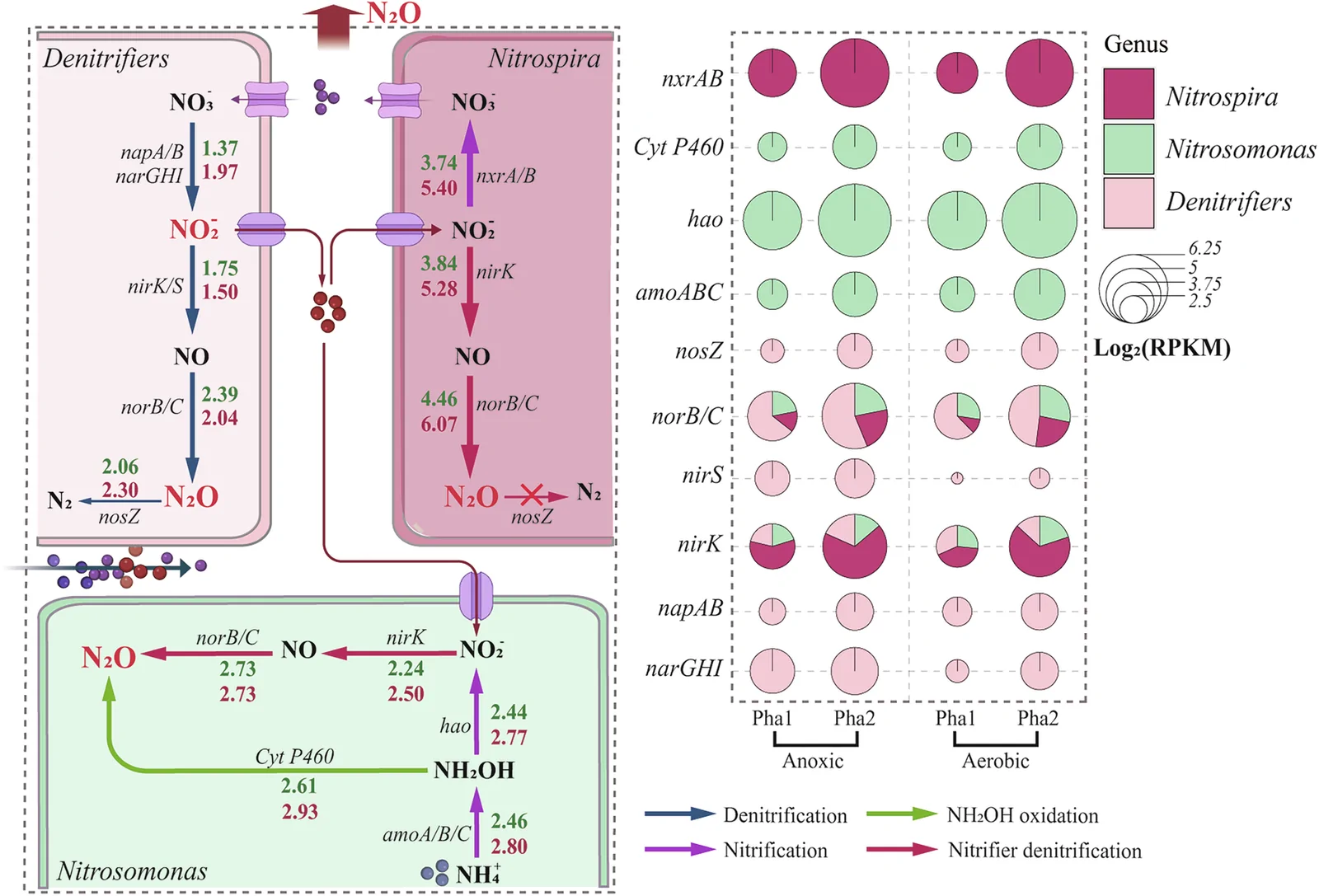
Putative N_2_O-production pathways and host-gene abundance in the anoxic and aerobic tanks inferred from metagenomics. Values next to genes refer to the fold-change of the specific gene in Phase 2 compared to Phase 1. Green character refers fold-change in the Anoxic tank. Red character refers fold-change in the Aerobic tank.

To further examine putative N_2_O-production potential in the Anoxic and Aerobic tanks, functional genes and their putative host taxa were analyzed (Fig. 4). AOB can produce N_2_O via hydroxylamine (NH_2_OH) oxidation using cytochrome P460 (*Cyt P460*) under high NH_4_^+^ and O_2_^29^. Nitrifier denitrification produces N_2_O using *nirK* under high NO_2_^−^ and low O_2_^30^. The third biotic pathway is by heterotrophic denitrifiers, where N_2_O is produced as an intermediate of denitrification^31^.

*Cyt P460*, primarily associated with *Nitrosomonas*, increased from 6.2 and 6.0 RPKM in Phase 1 to 16.0 and 17.6 RPKM in Phase 2, in the Anoxic and Aerobic tank, respectively, a 2.61- and 2.93-fold increase (Supplementary Data 3). Similarly, hydroxylamine production and consumption by *amoA/B/C* and *hao* increased proportionally by 2.46- and 2.44-fold (Anoxic) and 2.80- and 2.77-fold (Aerobic), respectively, in Phase 2 compared to Phase 1.

Genes associated with nitrifier denitrification also increased in Phase 2. *nirK* was assigned mainly to *Nitrosomonas* and *Nitrospira*. In *Nitrosomonas*, *nirK* increased by 2.24- and 2.50-fold in Phase 2 in the Anoxic and Aerobic tanks, respectively. In *Nitrospira*, *nirK* increased from 10.1 and 5.8 RPKM to 38.8 and 30.8 RPKM. Meanwhile, *norB/C* reducing NO to N_2_O increased by 2.73 ~ 6.07 times in *Nitrosomonas* and *Nitrospira* in the Anoxic and Aerobic tanks, respectively. Importantly, *nosZ* was not detected in either *Nitrosomonas* or *Nitrospira*.

Heterotrophic denitrification genes were also detected and increased in Phase 2. In denitrifier-assigned taxa, both *nirK* and *nirS* increased from 12.8 to 22.4 RPKM in the Anoxic tank and from 6.5 to 9.8 RPKM (1.50-fold) in the Aerobic tank. *napA/B*, *narGHI*, and *norB/C* also increased. For example, *norB/C* increased from 14.7 and 11.4 RPKM to 35.2 and 23.3 RPKM (2.39- and 2.04-fold) in the Anoxic and Aerobic tanks, respectively. *nosZ* also increased (from 4.5 and 4.3 RPKM in Phase 1 to 9.2 and 10.0 RPKM in Phase 2), but with relatively low abundance compared to other denitrification genes, indicating N_2_O accumulation potential by the denitrification pathway.

A supplementary *nirK/S* to *nosZ* ratio analysis further supported a higher relative representation of N_2_O-forming compared with N₂O-reduction potential in Phase 2 (Fig. S7 and Supplementary Data 4), but this ratio was interpreted only as qualitative ecological evidence rather than quantitative pathway partitioning.

**Fig. 5:**
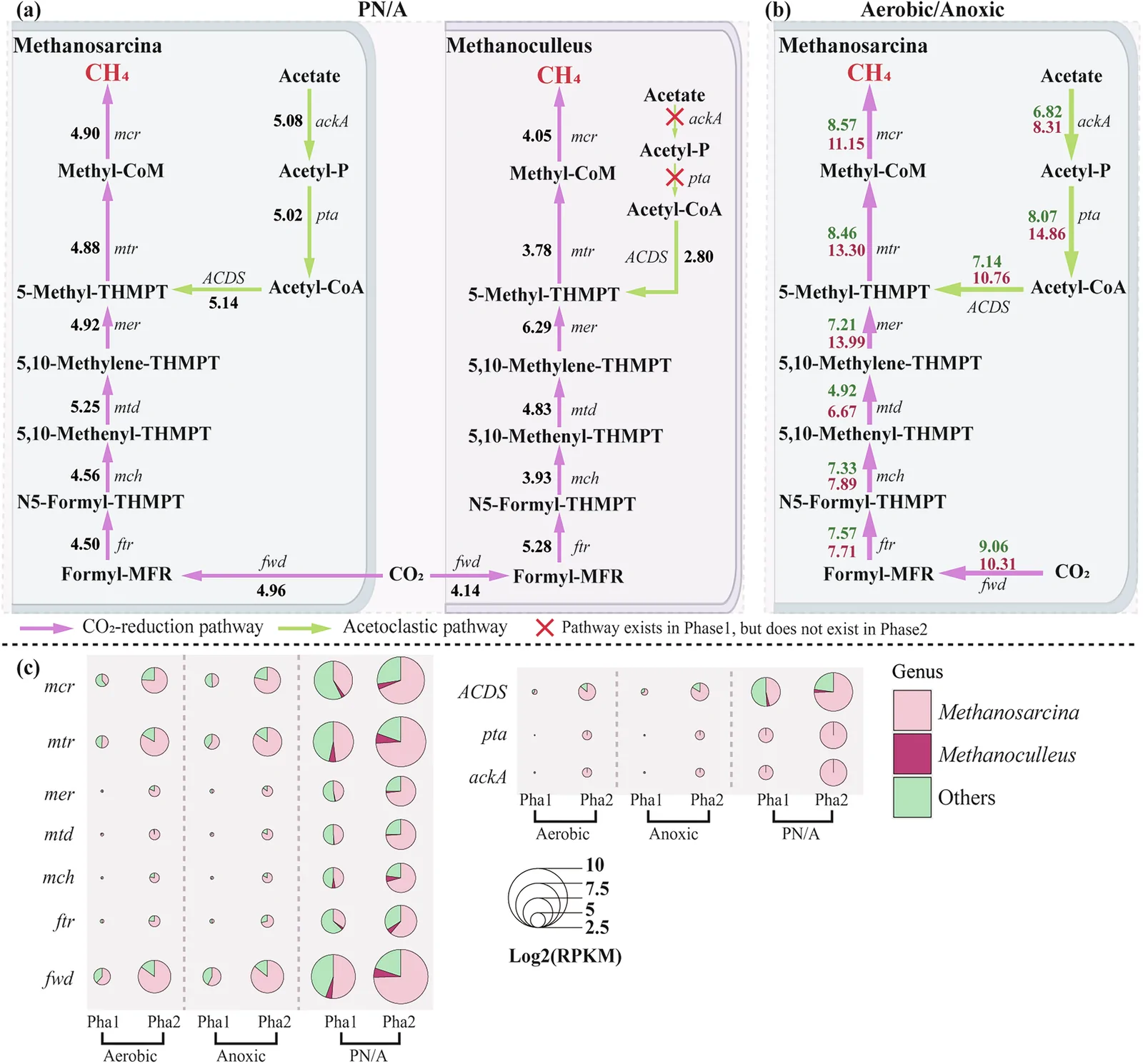
Putative methanogenesis pathways and relative host-gene abundance inferred from metagenomics. Model for putative CH_4_ production in the **a** PN/A tank, **b** Anoxic and Aerobic tanks. Values next to the genes refer to the fold changes in metagenomic gene relative abundance from Phase 1 to Phase 2. Green and red values refer fold-change in the Anoxic and Aerobic tanks, respectively. **c** methanogenesis-related gene abundance and assigned host genera. These gene data indicate methanogenic potential and do not provide a mathematical or quantitative relationship with CH_4_ emission rates.

Methanogenesis-related genes were examined to evaluate methanogenic functional potential across treatment units. CH_4_ production in WWTPs can occur via acetoclastic methanogenesis and the CO_2_-reduction/hydrogenotrophic methanogenesis (Fig. 5). In the AD unit, genes associated with both methanogenic pathways were detected, with a relatively higher number of genes affiliated with the hydrogenotrophic pathway than acetoclastic pathway genes (Table S4). Most methanogenesis-related genes decreased in relative abundance in Phase 2, including *fwd* (1077.5 to 679.8 RPKM), *mtr* (276.9 to 183.0 RPKM), and *mcr* (502.6 to 317.9 RPKM) (Fig. S6), whereas recovered CH_4_ increased. Routine monitoring showed that total VFAs in the digester-feed were higher in Phase 2 (6386.8 ± 628.2 mg/L) than in Phase 1 (5733.3 ± 639.9 mg/L), while VFAs in digested sludge remained low (319.0 ± 53.8 vs. 284.7 ± 22.1).

Genus-level assignment showed that methanogenesis-related genes in the AD unit were mainly associated with *Methanosarcina* and hydrogenotrophic methanogens, particularly *Methanobacterium*, whereas *Methanothrix*-associated genes were negligible (Table S4). In the PN/A tank, genes associated with both methanogenic pathways increased in Phase 2 (Fig. 5a, c). In the Anoxic and Aerobic tanks, methanogenesis-related genes were negligible in Phase 1 but increased markedly in Phase 2, mainly associated with *Methanosarcina* (Fig. 5b, c). Dissolved CH_4_ was not detected in the Anoxic tank and was detected only at low concentration in the Aerobic tank. ORP values were −96.6 to −23.4 mV in the Anoxic tank and +26.2 to +86.5 mV in the Aerobic tank (Fig. S8 and Table S5).

## Discussion

This full-scale observational study demonstrates that disturbance of side-stream PN/A can propagate beyond the side-stream reactor and affect mainstream treatment performance. It also highlights an operational trade-off between AD intensification and PN/A stability. Increasing externally imported sludge enhanced CH₄ recovery from AD but coincided with a higher C/N ratio in the PN/A tank, increasing from 1.39 in Phase 1 to 2.16 in Phase 2. Elevated C/N ratios have been reported to inhibit anammox activity, particularly when organic carbon promotes competition between heterotrophic organisms and AnAOB^32^. Consistent with this, PN/A ammonium removal efficiency declined from 87.6% to 55.8%, and specific anammox activity decreased sharply under disturbed operation.

Although the higher COD loading in Phase 2 could theoretically favor heterotrophic denitrification, effective denitrification also requires sufficient NO₂⁻ and/or NO₃⁻ availability^33^. In the disturbed PN/A tank, inhibition of AOB and AnAOB reduced partial nitritation and anammox activity, thereby limiting NO₂⁻/NO₃⁻ production. As a result, denitrification activity also declined, despite becoming relatively more important than anammox in Phase 2. The PN/A tank, therefore, shifted from an efficient partial nitritation–anammox system to a lower-efficiency system with reduced nitrification and anammox capacity.

The impaired PN/A process allowed more NH₄⁺ and COD to be recycled back to the mainstream Anoxic–Aerobic units through the return liquor. This increased the downstream nitrogen and organic burden, promoted mainstream nitrification, and contributed to higher NO₃⁻ accumulation in the Aerobic tank and final effluent. Consequently, apparent liquid-phase mainstream TN removal decreased from 472.4 to 298.2 kg-N/d, and final effluent quality deteriorated.

The GHG results should be interpreted within the monitoring boundary of this study. Dissolved N_2_O and CH_4_ were directly monitored only in the mainstream Anoxic and Aerobic tanks, and emissions from these tanks were estimated using mass-transfer-based calculations. In contrast, PN/A tank N_2_O emissions were estimated using emission factors because dissolved N_2_O was not directly monitored in the PN/A reactor. Fugitive CH_4_ losses from the AD unit and GHG emissions associated with the final effluent were also not measured. Therefore, this study supports a comparison of directly monitored mainstream dissolved-gas-derived emission estimates, supplemented by factor-based side-stream estimates and biogas-electricity offset, rather than a fully resolved plant-scale GHG budget. Within this boundary, disturbed PN/A operation was associated with higher estimated mainstream N₂O and CH₄ emissions and a higher expanded plant-level climate burden.

The microbial community and functional-gene results provide ecological context for the process-level deterioration observed during disturbed PN/A operation. In the AD unit, enrichment of hydrolytic, acidogenic, and acetogenic bacteria was consistent with increased substrate throughput under higher sludge loading^34^. THP can enhance sludge solubilization and increase the availability of fermentable substrates, thereby favoring fermentative populations and altering methanogenic community structure^35,36^. The lower relative abundance of methanogens in Phase 2 should therefore be interpreted as a community-structure shift associated with changed substrate supply and higher sludge throughput, rather than as direct evidence of methanogenic inhibition. Consistently, the higher recovered CH₄ in Phase 2 likely reflected increased organic loading and substrate conversion at the whole-digester scale, whereas DNA-based relative abundance does not quantify absolute methanogen biomass or in situ methanogenic activity.

In the PN/A tank, the loss of *Ca. Brocadia* and the near-disappearance of *Nitrosomonas*, together with strong depletion of anammox and partial-nitrification genes, were consistent with the observed decline in anammox activity and PN/A ammonium removal. This community shift likely reflected the elevated COD and C/N ratio in the PN/A tank, which can favor heterotrophic populations and intensify competition with AnAOB and AOB^37,38^. As a result, the disturbed PN/A system produced less NO₂⁻/NO₃⁻ through partial nitritation and anammox, reducing nitrogen removal efficiency and allowing more NH₄⁺ and COD to return to the mainstream.

In the downstream Anoxic and Aerobic tanks, enrichment of *Nitrosomonas* and *Nitrospira* was consistent with higher NH₄⁺ transfer from the disturbed PN/A system and enhanced downstream nitrification, helping explain the elevated NO₃^−^ concentrations in both mainstream tanks^39,40^. Although denitrifier-associated taxa also increased, the higher NO_3_^−^ concentrations suggest that NO_3_^−^ production likely exceeded NO_3_^−^ reduction under Phase 2 operational conditions. This imbalance may have reflected the higher upstream nitrogen burden, carbon availability, hydraulic conditions, and the difference between DNA-level functional potential and active pathway rates.

The N₂O-related gene patterns further suggest increased nitrifier-associated N₂O-production potential in the Anoxic and Aerobic tanks. Increased *Nitrosomonas*-associated cytochrome P460 was consistent with greater hydroxylamine-turnover potential, while higher *nirK* and *norB/C* abundances assigned to *Nitrosomonas* and *Nitrospira* were consistent with increased nitrifier-denitrification potential^29,30^. These patterns provide a plausible ecological context for the higher dissolved N₂O concentrations and dissolved-gas-based mainstream N₂O emission estimates. However, they do not demonstrate that these pathways directly drive N₂O emissions under plant conditions. Heterotrophic denitrification remains a plausible contributor, particularly because the rbCOD of the combined mainstream influent and the off-line SDNR of mainstream sludge were not measured. Therefore, the metagenomic data should be interpreted as evidence of functional potential and likely microbial contributors, rather than as quantitative source partitioning of N₂O flux.

The methanogenic results should be interpreted by distinguishing whole-digester CH₄ recovery from DNA-level relative gene abundance. THP sludge digestion can increase sludge solubilization and alter substrate availability, and previous studies have reported that hydrogenotrophic methanogenesis may become more important under pretreated sludge digestion^41^. In this study, methanogenesis-related genes in the AD unit were mainly associated with the metabolically versatile *Methanosarcina* and hydrogenotrophic methanogens such as *Methanobacterium*, whereas *Methanothrix*-associated genes were negligible. This pattern suggests that the AD methanogenic community was not dominated by obligate acetoclastic *Methanothrix*^42,43^, although gene abundance alone cannot quantitatively determine pathway dominance.

The apparent discrepancy between increased recovered CH₄ and decreased relative abundance of methanogenesis-related genes likely reflects the difference between whole-digester performance and microbial relative abundance. Routine monitoring showed higher total VFAs in the digester feed in Phase 2, while VFAs in digested sludge remained low in both phases, indicating substantial VFA conversion during digestion. Therefore, the higher recovered CH₄ in Phase 2 was more likely associated with increased substrate loading and conversion through the AD process than with a higher relative abundance of methanogenesis genes. Because the metagenomic data are DNA-based and relative, they do not quantify absolute methanogen abundance, gene expression, enzymatic activity, or methane-production rates.

In the mainstream tanks, the increase in *Methanosarcina* and methanogenesis-related genes should also be interpreted cautiously. Dissolved CH₄ was not detected in the Anoxic tank and was detected only at low concentration in the Aerobic tank, while ORP values did not indicate strongly methanogenic bulk-liquid conditions. Thus, the detected methanogenic signatures may reflect biomass carry-over from upstream sludge or side-stream units, persistence within micro-anoxic sludge-floc niches, or release from biomass, rather than confirmed active bulk-liquid methanogenesis in the mainstream tanks.

Several limitations should be considered. First, because sludge-associated nitrogen, gaseous nitrogen losses, and storage terms were not measured, only an apparent liquid-phase mainstream TN balance could be calculated. Second, rbCOD and VFA in each tank were not directly measured, and off-line specific denitrification rate tests were not conducted for mainstream sludge. Therefore, the relative contributions of nitrifier-associated pathways and heterotrophic denitrification to N_2_O production could not be quantitatively partitioned. Third, PN/A activity tests were conducted only with PN/A fresh sludge under controlled laboratory conditions and therefore represent potential activity rather than direct in-line pathway-specific rates. In addition, PN/A internal recirculation rates during both phases were not recorded, which further limited quantitative evaluation of denitrification in the PN/A tank. Finally, metagenomic data describe DNA-level relative abundance and functional potential, but do not quantify absolute microbial abundance, gene expression, enzyme activity, or in situ transformation rates.

Future studies should combine direct dissolved and off-gas monitoring across all treatment units, including the PN/A reactor and AD gas system, with AD biogas leakage quantification. Integration of metatranscriptomics, proteomics, absolute quantification of key functional groups, rbCOD/VFA measurements, specific denitrification tests, and full nitrogen mass balance would further resolve active microbial pathways and improve plant-scale GHG accounting. From an operational perspective, the results indicate that AD intensification for biogas recovery should be evaluated together with PN/A stability and mainstream GHG emissions, rather than optimized as an isolated unit process.

## Methods

### Process description and sampling strategy

**Fig. 6:**
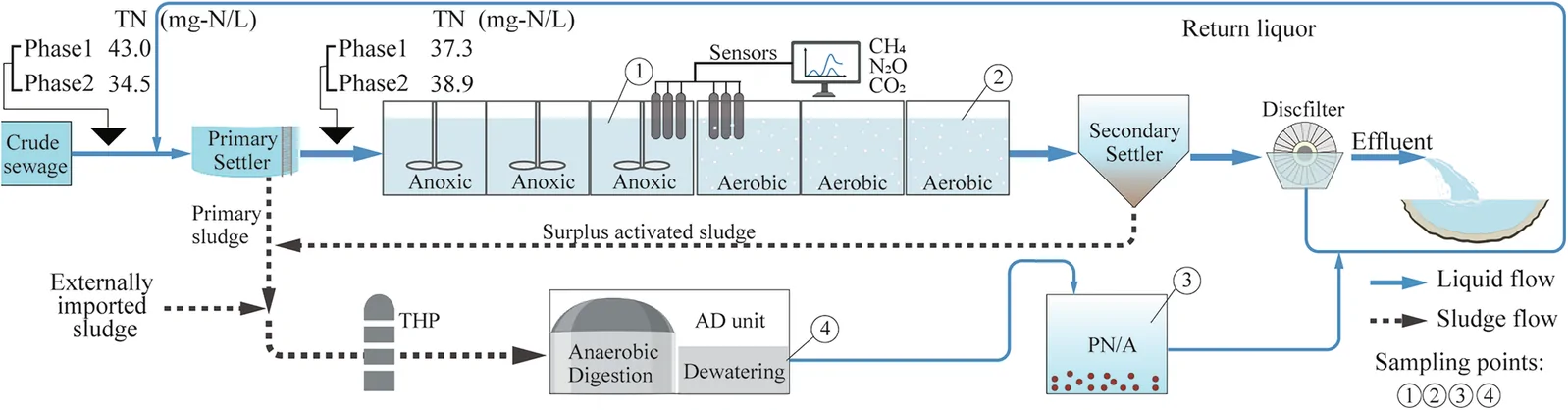
Process configuration and sampling locations of the anoxic-aerobic-AD-PN/A WWTP. Phase 1: stable PN/A operation, Phase 2: disturbed PN/A operation. The first TN label represents crude sewage TN; the second TN label represents the mainstream influent after mixing of crude sewage with returned liquors. Anoxic-Aerobic process serves as the mainstream treatment unit, and PN/A serves as the side-stream treatment unit. THP: Thermal Hydrolysis Process, and an AD unit for biogas recovery.

The municipal WWTP serves a population equivalent of approximately 140,000 in the UK. It employs a conventional Anoxic/Aerobic process for mainstream wastewater treatment and a side-stream PN/A system for AD dewatering liquor treatment (Fig. 6). The PN/A effluent is combined with mainstream disc-filter backwash and returned to the mainstream. A thermal hydrolysis pretreatment (THP) unit is installed upstream of the AD process to enhance sludge hydrolysis and biogas production. The THP receives indigenous primary sludge from the primary settler and surplus activated sludge (SAS) from the secondary settler, together with imported co-settled primary and humus sludge from percolating filter works and SAS from smaller activated sludge plants. The influent crude sewage is dosed with ferric sulfate primarily for chemical phosphorus removal and to aid coagulation and is then mixed with return liquors before entering the mainstream biological treatment process. The dimensions and operational parameters of the Anoxic, Aerobic, and PN/A tanks are listed in Table S5. The designed THP feeding is approximately 45 tDS/d, comprising around 15 tDS/day indigenous sludge and 30 tDS/d imported sludge. During the study period, the dissolved oxygen (DO), pH, and ORP in the mainstream Anoxic and Aerobic tanks remained within stable operational ranges (Fig. S8).

The investigation was conducted in two operational phases. Phase 1 represents satisfied PN/A performance (March 2023), while Phase 2 represents the disturbed PN/A (August 2023) (Table S6). Although the influent wastewater flow to the WWTP did not increase in Phase 2, the AD unit experienced increased organic loading due to a deliberate increase in externally imported sludge, from 263.4 m^3^/day in Phase 1 to 462.9 m^3^/day in Phase 2 (Fig. S9a), to maximize CH_4_ recovery. As a result, the organic loading rate (OLR) of the AD increased from approximately 2.15 to 3.35 kg VS/m^3^/day. This increase was accompanied by higher CH_4_ production and electricity generation (Figs. 2a and S9b).

Crude sewage TN decreased from 43.0 mg-N/L in Phase 1 to 34.5 mg-N/L in Phase 2, and COD increased slightly from 524.2 to 549.0 mg/L (Fig. S2b, c). In contrast, TN in the combined mainstream influent, after mixing of crude sewage with return liquors, increased from 37.3 to 38.9 mg-N/L (Figs. S2d and 6). This opposite trend indicates that the higher mainstream influent TN concentration in Phase 2 was not driven by crude sewage quality, but was likely associated with increased nitrogen recycling through return liquors under disturbed PN/A operation. Meanwhile, the mainstream influent flow decreased from 41,439 m^3^/day in Phase 1 to 34,277 m^3^/day in Phase 2 (Fig. S2a), indicating reduced COD and TN loading in Phase 2.

Wastewater samples were collected in triplicate, and sludge samples in duplicate, weekly over three consecutive weeks in each phase. Samples were taken from the Anoxic, Aerobic, AD unit, and PN/A tank (Fig. 6) and transported to the lab in a cooler box with ice packs. All filtered wastewater samples were analyzed immediately upon arrival in the laboratory. Samples were filtered using a membrane filter (0.45 μm, Merck). Sludge samples for specific activity tests were collected in sterile 250 mL bottles. Sludge samples for DNA extraction were collected in sterile 50 mL centrifuge tubes and stored at −20 °C upon arrival in the lab.

### Chemical and gas analysis

Filtered wastewater samples were analyzed for NH_4_^+^-N, NO_2_^−^-N, NO_3_^−^-N, and COD concentrations using Spectroquant test kits (Supelco, Merck) according to the manufacturer’s protocol. Dissolved CH_4_, CO_2_, and N_2_O concentrations were continuously monitored using in-situ probes installed in the Anoxic and Aerobic tanks, with an auto-sampling interval of 15 s. Dissolved CH_4_ and CO_2_ were measured using Swale probes with measurement ranges of 0.00–10.00 mg/L and 0.00–50.00 mg/L, respectively, while dissolved N_2_O was measured using a Unisense probe with a measurement range of 0.0–1.5 mg/L.

### Specific activity tests

Specific anammox, denitrification, and nitrification activities were assessed using sludge samples from the PN/A tank. Specific activity tests were conducted in 250 mL serum bottles with a working volume of 200 mL. Each bottle was inoculated with 3 g of centrifuged sludge and fed with synthetic wastewater (Table S7). For anammox and denitrification activity tests, the medium was flushed with nitrogen gas for 20 min to establish anaerobic conditions, and serum bottles were maintained in an anaerobic chamber. For the nitrification activity test, bottles were aerated to maintain aerobic conditions.

### DNA extraction, shotgun metagenomic sequencing, and data analysis

Frozen sludge samples (Table S8) from the four tanks were thawed on ice and centrifuged at 4000 × *g* for 10 min. The supernatant was discarded, and the pellet was used for DNA extraction. For each sampling week, duplicate sludge samples were processed separately for DNA extraction using the DNeasy PowerSoil Pro Kit (QIAGEN, UK) following the manufacturer’s protocol and subsequently pooled in equal mass prior to sequencing. As a result, three pooled DNA samples per tank (one per week) were obtained for metagenomic sequencing. DNA extract concentration and purity were assessed using a Qubit fluorometer (ThermoFisher, UK) and a 1% agarose gel electrophoresis (E-Gel^TM^ Power Snap Electrophoresis System, ThermoFisher), respectively. Qualified DNA samples were submitted to shotgun sequencing on an Illumina platform (PE150 mode) at Novogene Bioinformatics Technology (UK).

The raw sequencing data were processed following the pipeline described in Wang et al.^22^. Briefly, raw reads were quality-filtered and trimmed using Trimmomatic (v0.40). High-quality reads were assembled into contigs using MEGAHIT (v1.2.9), and contigs shorter than 200 bp were removed. Open reading frames (ORFs) were predicted from assembled contigs using Prodigal, while rRNA genes were identified using Barrnap. Functional annotation of predicted ORFs was performed against the KEGG database using DIAMOND. To identify key enzymes involved in AnAOB nitrogen metabolism that are not well represented in KEGG, additional annotations were conducted using the NCycDB database based on HMM homology searches.

### GHG emissions estimation

N_2_O emissions from the Anoxic and Aerobic tanks, and CH_4_ emissions from the Aerobic tank, were estimated from in situ dissolved-gas concentrations using published gas-liquid transfer models for wastewater treatment systems. This approach assumes that dissolved gases present in the mixed liquor are emitted to the atmosphere according to reactor-specific mass-transfer conditions^44^. For the aerobic tank, N_2_O transfer was estimated using an aeration-driven gas-stripping model that incorporates airflow, Henry’s law partitioning, reactor volume, and the N₂O mass-transfer coefficient. For the anoxic tank, N_2_O transfer was estimated using a non-aerated mass-transfer model driven by the concentration gradient between measured dissolved N_2_O and atmospheric equilibrium. Site-specific operational parameters, including temperature, airflow, reactor volume, tank depth, superficial gas velocity, and power input, were used for the calculations. For the PN/A tank, dissolved N_2_O was not directly monitored; therefore, N_2_O emissions were indirectly estimated using TN-removal-based emission factors from the cited literature and Intergovernmental Panel on Climate Change (IPCC) guidance.

Aerobic tank:

1 $${E}_{{{\rm{N}}}_{2}{\rm{O}}}={H}_{{{\rm{N}}}_{2}{\rm{O}}}\times {C}_{{{\rm{N}}}_{2}{\rm{O}}}\left(1-{e}^{-\frac{{K}_{{\rm{L}}}{\alpha }_{{{\rm{N}}}_{2}{\rm{O}}}}{{H}_{{{\rm{N}}}_{2}{\rm{O}}}}\times \frac{{V}_{{\rm{R}}}}{{Q}_{{\rm{A}}}}}\right)\times \frac{{Q}_{{\rm{A}}}}{{V}_{{\rm{R}}}}$$

Anoxic tank:

2 $${E}_{{{\rm{N}}}_{2}{\rm{O}}}={K}_{L}{\alpha }_{{N}_{2}O,{T}_{Process}}^{non-aerated}\times \left({C}_{{N}_{2}O}-\frac{{{C}_{N}}_{2}O,air}{{H}_{{N}_{2}O}}\right)$$

Where:

${E}_{{{\rm{N}}}_{2}{\rm{O}}}$, N_2_O emissions rate (kg/day),

${H}_{{{\rm{N}}}_{2}{\rm{O}}}$, Henry’s law constant,

$${C}_{{{\rm{N}}}_{2}{\rm{O}}}$$, N_2_O dissolved concentration (g-N/m^3^),

*Q*_A_, total airflow through the tank (m^3^/d),

*V*_R_, the volume of the aerated part of the reactor (m^3^),

${K}_{{\rm{L}}}{\alpha }_{{{\rm{N}}}_{2}{\rm{O}}}$, N_2_O mass transfer coefficient (d^−1^),

${{\rm{K}}}_{L}{\alpha }_{{{\rm{N}}}_{2}{\rm{O}},{T}_{\mathrm{Process}}}^{\mathrm{non}-\mathrm{aerated}}$, N_2_O mass transfer coefficient in non-aerated zones (d^−1^),

${C}_{{{\rm{N}}}_{2}{\rm{O}},\mathrm{air}}$, N_2_O concentration in air (g-N/m^3^).

At an average temperature of 13 °C, ${H}_{{{\rm{N}}}_{2}{\rm{O}}}$ was 0.025. The ${K}_{L}{\alpha }_{{{\rm{N}}}_{2}{\rm{O}},\,{T}_{\mathrm{Process}}}^{\mathrm{non}-\mathrm{aerated}}$ value was set to 3 d^−1^, a typical value for an anoxic tank^45^. The average atmospheric N_2_O concentration in the Northern Hemisphere is 0.0003 g-N/m^3^^44,46^.

3 $${K}_{L}{\alpha }_{{{\rm{N}}}_{2}{\rm{O}}}={K}_{L}{\alpha }_{{N}_{2}O\,20^\circ C}\times {1.024}^{({T}_{Process}-20)}$$

4 $${K}_{L}{\alpha }_{{N}_{2}O\,20^\circ C}={\left(\frac{{D}_{R}}{{D}_{L}}\right)}^{-0.49}\times 34,500\times {\left({v}_{g}\right)}^{0.86}$$

*D*_R_, depth over the diffuser of the tank (m)^44^.

*D*_L_, depth of the initial laboratory reactor = 0.815 m^44^.

*v*_g_, the superficial gas velocity of the reactor (m/s)^44^. On-site information and constants were included in Table S9.

Regarding CH_4_ emissions in aerobic tank,

5 $${E}_{C{H}_{4}}={K}_{L}{\alpha }_{C{H}_{4}}\times \left({C}_{C{H}_{4}}-{C}_{C{H}_{4}}^{* }\right)$$

Where:

${E}_{{\mathrm{CH}}_{4}}$, CH_4_ emissions rate (kg/day),

${K}_{L}{\alpha }_{{\mathrm{CH}}_{4}}$, CH_4_ mass transfer coefficient (h^−1^),

${C}_{{\mathrm{CH}}_{4}}$, dissolved CH_4_ concentration (g/m^3^),

${C}_{{\mathrm{CH}}_{4}}^{* }$, equilibrium CH_4_ concentration (g/m^3^)^47,48^

6 $${K}_{L}{\alpha }_{C{H}_{{4}}}=0.86\times {K}_{L}{\alpha }_{{O}_{2}}$$

7 $${K}_{L}{\alpha }_{{O}_{2}}=0.2\times {\left({P}_{g}\right)}^{0.5}\times {\left({v}_{g}\right)}^{0.2}$$

${K}_{L}{\alpha }_{{{\rm{O}}}_{2}}$, O_2_ mass transfer coefficient (h^−1^)^47,48^.

$${P}_{g}$$, power input (W/m^3^)^47,48^.

CH_4_ concentration in the aerobic tank was negligible compared to CO_2_ (Fig. 2c, d), allowing the assumption of ${C}_{{{CH}}_{4}}^{* }$ to be negligible (≈0)^49^. In the Anoxic tank, CH_4_ remained undetectable in both operational phases and was therefore excluded from calculations. On-site information and constants are shown in Table S9.

In the PN/A tank, dissolved N_2_O was not measured and was therefore estimated based on the IPCC Guidelines^5,50^.

8 $$E{m}_{\left(PN/A\right)}=0.0062\times TN\_Remov{e}_{(PN/A)}$$

9 $$E{m}_{\left(Dn\right)}=0.016\times TN\_Remov{e}_{(Dn)}$$

10 $$E{m}_{\left(Total\right)}=E{m}_{\left(PN/A\right)}+E{m}_{\left(Dn\right)}$$

Where:

Em_(PN/A),_ Em_(Dn),_ and Em_(Total)_ refer to N_2_O emissions from the PN/A pathway, denitrification pathway, and total emissions from the PN/A tank, respectively.

CO_2_ equivalent (CO_2_-eq) calculation:

11 $$eq.C{O}_{2}=298\times {E}_{{N}_{2}O}+28\times {E}_{C{H}_{4}}$$

These calculations provide model-based estimates rather than direct off-gas measurements. Therefore, they were used to compare relative emission changes between the two operational phases under the same plant configuration, rather than to construct a fully resolved plant-scale GHG inventory.

## Supplementary information

Supplementary Information

Supplementary Data

## Data Availability

Raw shotgun metagenomic sequencing data generated in this study have been deposited in the European Nucleotide Archive under study accession PRJEB113830 and will be publicly available upon publication. Operational plant datasets are not publicly available due to confidentiality agreements with the collaborating full-scale wastewater treatment plant, but are available from the corresponding author on reasonable request and subject to permission from the plant operator.
