# Supplementary material for: Propagation of side-stream partial nitritation–anammox disturbance to mainstream wastewater greenhouse gas emissions
Source: NPJ Clean Water. 2026 Jun 24;9(1):75. doi: 10.1038/s41545-026-00600-8 (PMC13658029; doi:10.1038/s41545-026-00600-8)
Supplement: Supplementary file 1 [file 41545_2026_600_MOESM1_ESM.pdf]

Supplementary Information

Propagation of side-stream partial nitritation–anammox disturbance to mainstream wastewater greenhouse gas emissions

Zhufang Wang[a], Xiaobo Shen[a,b], Bang Du[a], Wenhan Li[c], Paul Church[b], Claudio Avignone-Rossa[d], Kai Yang[e], Bing Guo[a,*]

[a] School of Engineering, University of Surrey, GU2 7XH, Guildford, UK

[b] Thames Water Utilities Ltd, Clearwater Court, Vastern Road, RG1 8DB, Reading, UK

[c] Department of Mathematics, University of York, YO10 5DD, York, UK

[d] Microbes, Infection and Immunity Division, University of Surrey, Guildford, GU2 7XH, UK

[e] Advanced Technology Institute, University of Surrey, Guildford, GU2 7XH UK

* Corresponding author.

E-mail address: b.guo@surrey.ac.uk (B. Guo)

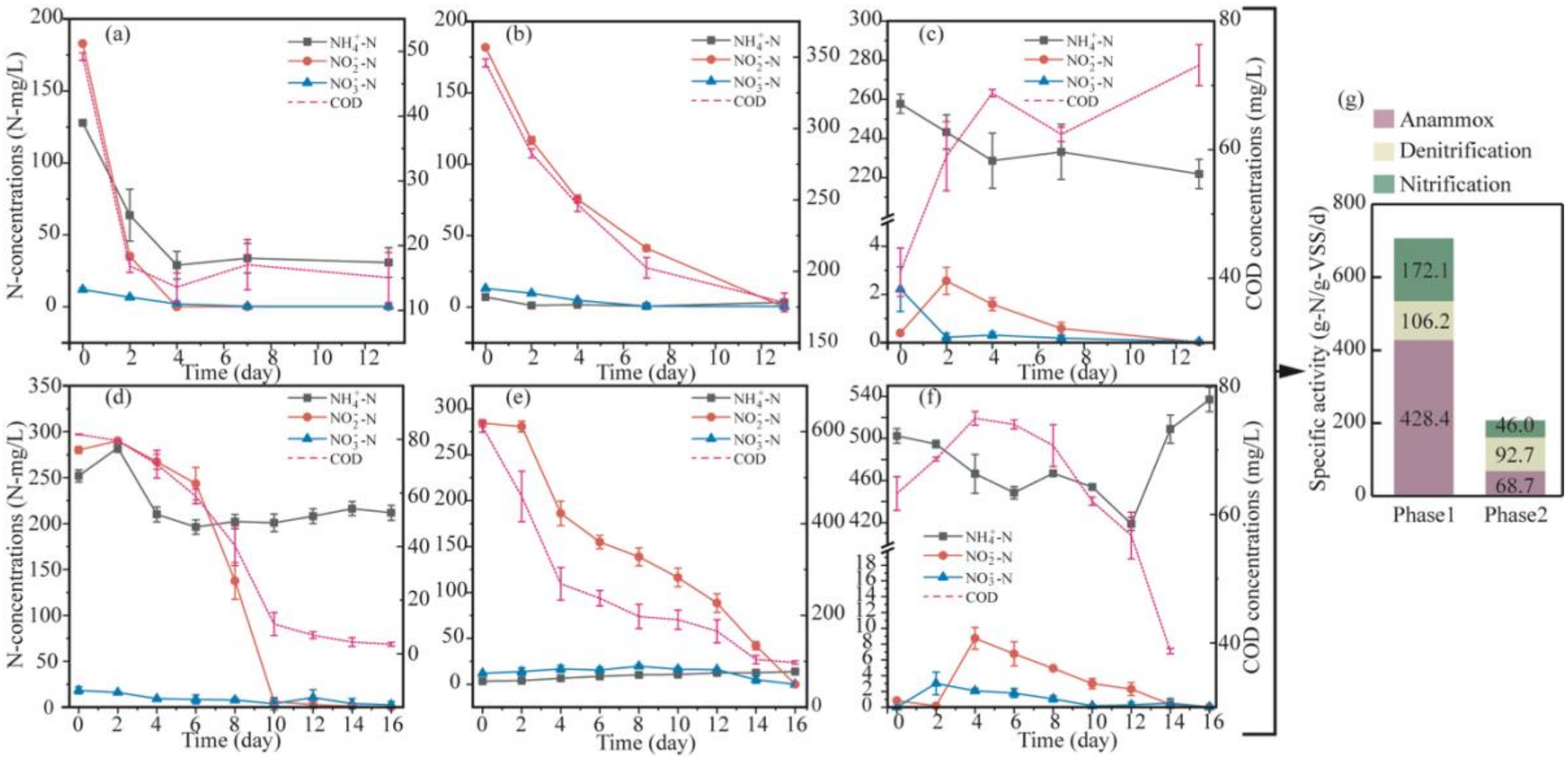

Fig. S1 Specific activity tests. (a)~(c): activity of anammox, denitrification, and nitrification in Phase1, respectively. (d)~(f): activity of anammox, denitrification, nitrification in Phase2, respectively. (g) comparison of specific activity test between two phases.

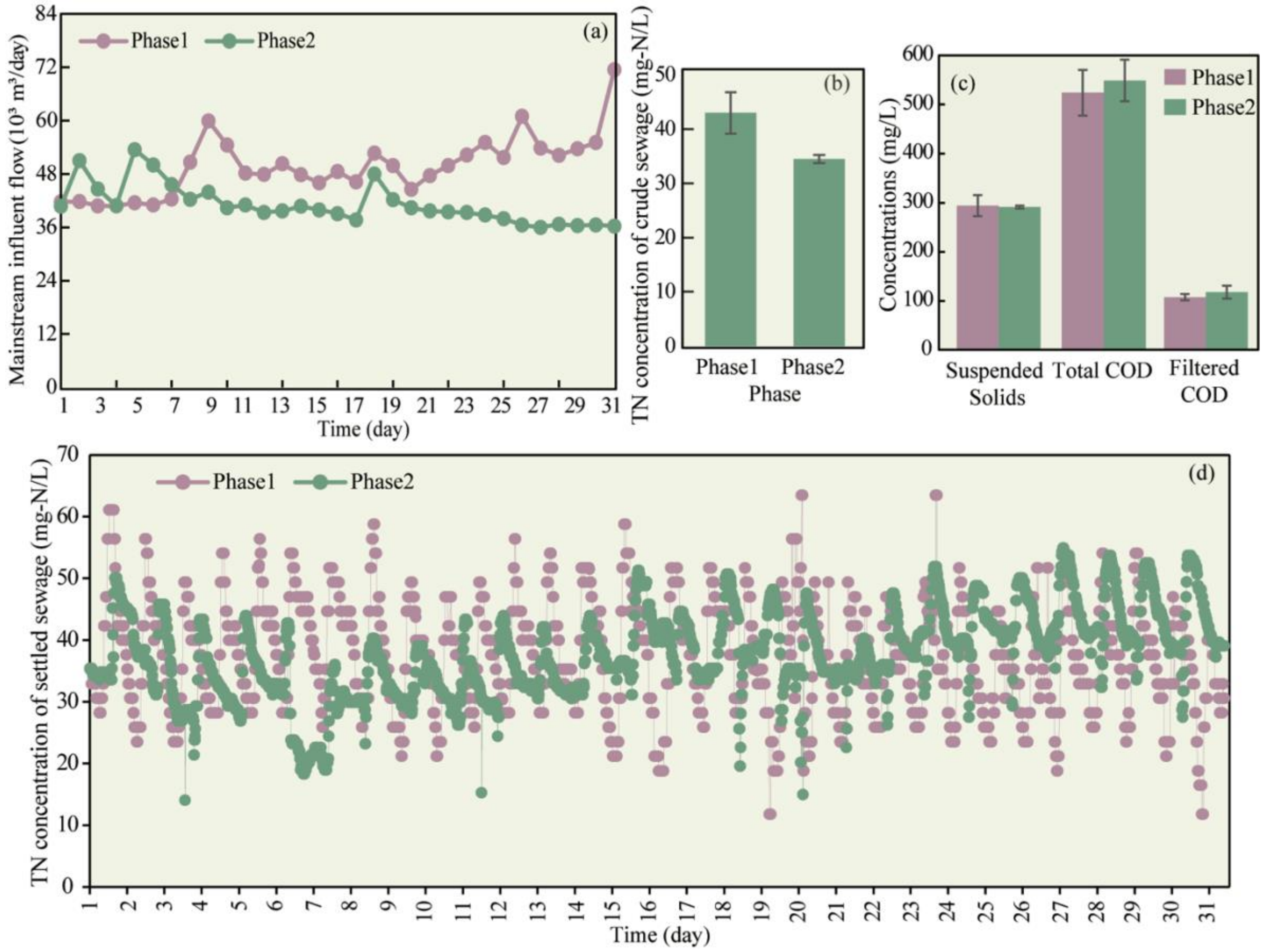

Fig. S2 Nitrogen concentration and flow rate of crude sewage and mainstream influent. (a) Mainstream influent flow. (b) TN concentration of crude sewage in two phases. (c) Suspended solids, Total COD, filter COD concentration of crude sewage. (d) TN concentration of settled sewage in two phases. For each phase in (a) and (d), 30 days of data are presented. Data averaged over one month were used for comparison between two phases.

At the kingdom level, the relative abundance of archaea decreased from 27.4 ± 5.9% in Phase1 to 18.0 ± 4.3% in Phase2 (Fig. S3) in the AD unit. In contrast, the PN/A tank increased from 24.3 ± 8.0% in Phase1 to 28.7 ± 0.9% in Phase2). In the Anoxic and Aerobic tanks, archaea abundance in Phase2 was 8.8-fold and 11.1-fold higher, respectively, compared to Phase1. Clearly, the AD unit and disturbed PN/A provided a source of archaea population to the mainstream units.

At the phylum level, the AD unit was dominated by Firmicutes, Euryarchaeota, and Atribacterota (Fig. S3). From Phase1 to Phase2, Firmicutes increased (43.6 ± 5.3% to 52.0 ± 4.8%), while Euryarchaeota (27.4 ± 6.0% to 18.0 ± 4.3%) and Atribacterota (20.0 ± 3.0% to 16.7 ± 3.7%) declined. In the PN/A tank, Planctomycetes and Proteobacteria declined sharply from 17.9 ± 2.2% and 13.1 ± 5.1% in Phase1 to 0.57 ± 0.03% and 7.2 ± 2.2% in Phase2, respectively. Conversely, in the Anoxic and Aerobic tanks, Proteobacteria increased by 16.1 ± 3.1% and Nitrospirae by 17.8 ± 3.3% from Phase1 to Phase2.

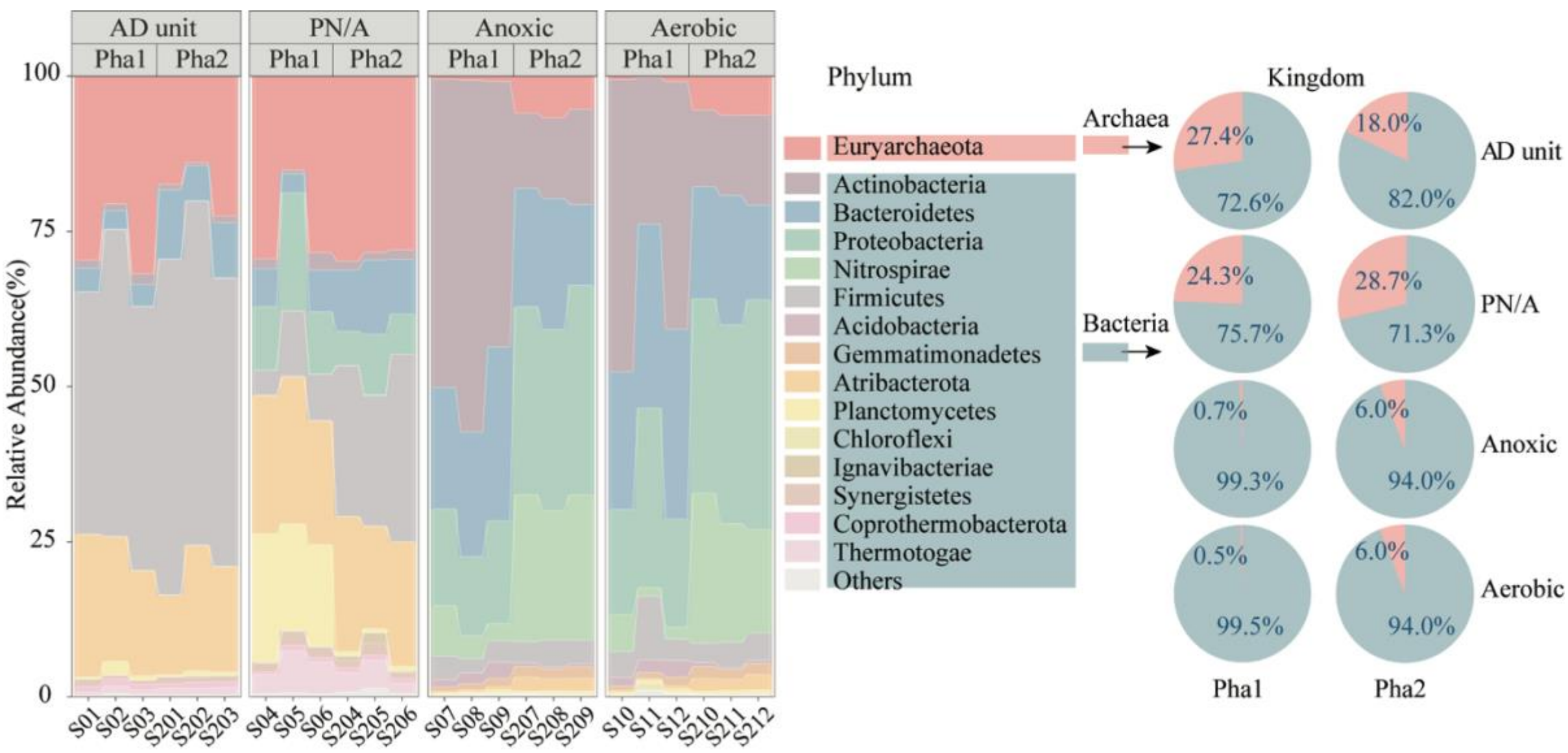

Fig. S3 Microbial community composition at kingdom and phylum levels across treatment units and operational phases. The stacked bar plot shows the relative abundance of dominant microbial phyla in the AD unit, PN/A tank, Anoxic tank, and Aerobic tank during Phase1

(Pha1) and Phase2 (Pha2). Each bar represents an individual sample, and taxa with low relative abundance are grouped as “Others”. The pie charts show the relative proportions of Archaea and Bacteria at the kingdom level in each treatment unit and phase. Pha1: Phase 1; Pha2: Phase 2.

For functional profiling, predicted genes were annotated against the KEGG database, and assigned to metabolic pathways at levels 1-3 based on KEGG Orthologs (KOs) (Fig. S4). The relative abundance of each pathway was calculated as the sum of RPKM-normalized abundances of KOs assigned to that pathway, divided by the total RPKM-normalized abundance of all annotated KOs within each sample and domain (bacteria or archaea), and expressed as a percentage. In the AD unit, reactions by archaea decreased significantly from 24.4% in Phase1 to 15.7% in Phase2 ($p<0.05$), while bacteria (>75%) dominated in both phases (Fig. S4a). In contrast, archaeal reactions increased in the downstream three tanks during Phase2, consistent with the observed shifts in microbial community composition. At KEGG level 1, Metabolism was the most abundant group (>95%) in all tanks for both archaea and bacteria (Fig. S4b&c). At level 2, the top pathways included Energy metabolism, Carbohydrate metabolism, and Metabolism of cofactors and vitamins (Fig. S4d&e). These pathways are critical for organic matter degradation, energy production, and nitrogen metabolism, reflecting the robust metabolic capabilities of the microbial communities in the WWTP.[1]

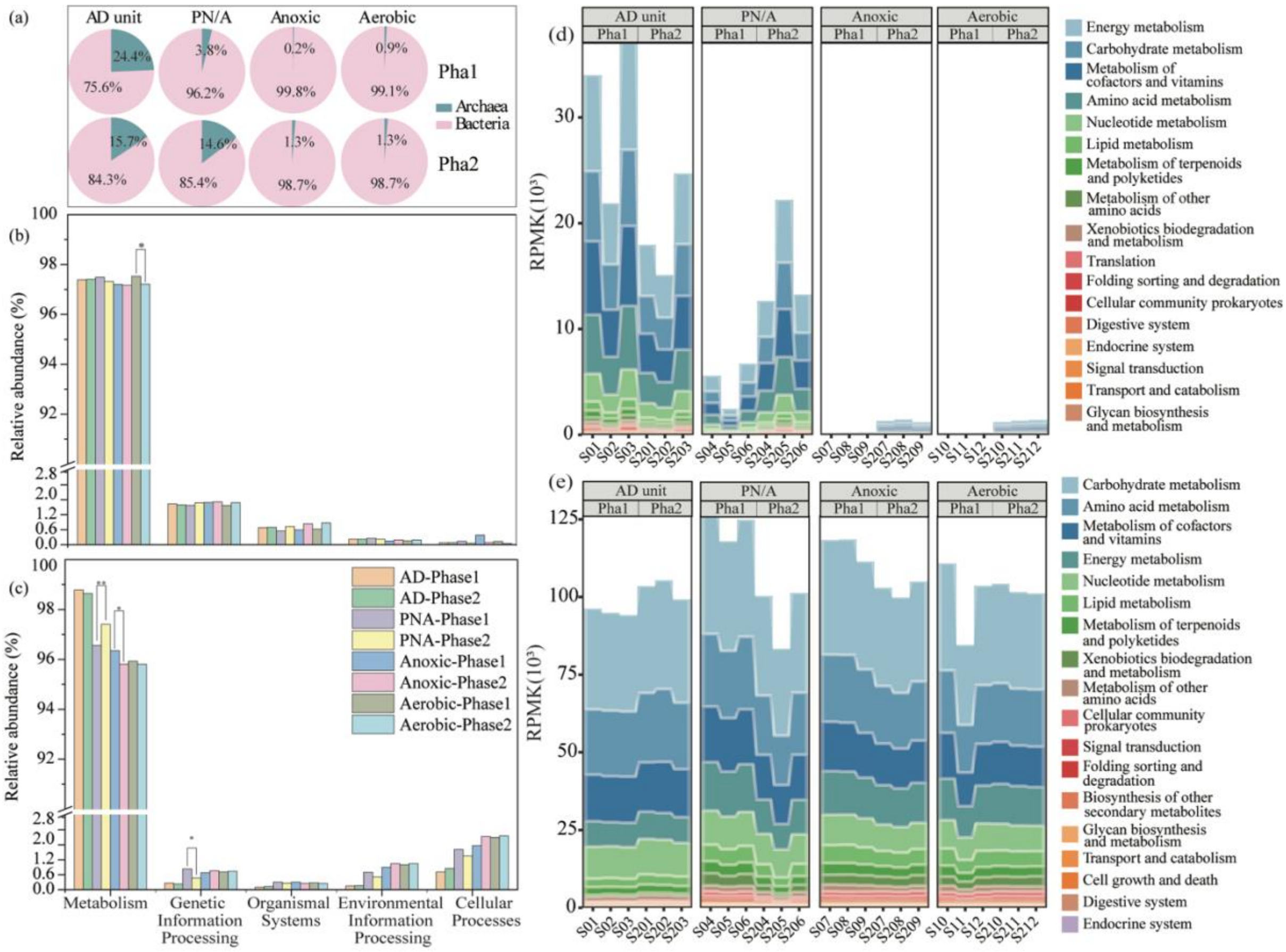

Fig. S4 Relative abundance of KEGG metabolic pathways at level-1 and level-2. (a) carried out by archaea and bacteria. (b) at level-1 carried out by archaea. (c) at level-1 carried out by bacteria. (d) at level-2 carried out by archaea. (e) at level-2 carried out by bacteria. Statistical comparisons were based on n=3 independent samples per phase.

At level 3, for archaea, Methane metabolism (KEGG:00680) pathways involved in methane metabolism dominated in the AD unit (17.18% - 19.82%) (Fig. S5a), aligning with its role in CH_4 production.[2] In Phase2, methane metabolism increased in the downstream three tanks, suggesting enhanced methanogenic potential and CH_4 emissions. Conversely, oxidative phosphorylation and tricarboxylic acid (TCA) cycle activity decreased in the anoxic and aerobic tanks, indicating a shift towards anaerobic metabolism. For bacteria, Oxidative phosphorylation (3.62% - 4.92%, KEGG:00190) and Glycolysis (3.51% - 5.86%, KEGG:00010) were the most abundant KEGG level 3 pathways (Fig. S5b). Compared to Phase 1, glycolysis increased in the PN/A tank but decreased in the Anoxic and Aerobic tanks, while nitrogen metabolism decreased in the PN/A tank but increased in the Anoxic and Aerobic tanks, and oxidative phosphorylation decreased in all three tanks in Phase 2. These results indicate a metabolic shift towards anaerobic pathways in the downstream tanks, driven by the disturbed PN/A process and the resulting changes in PN/A effluent composition.[3]

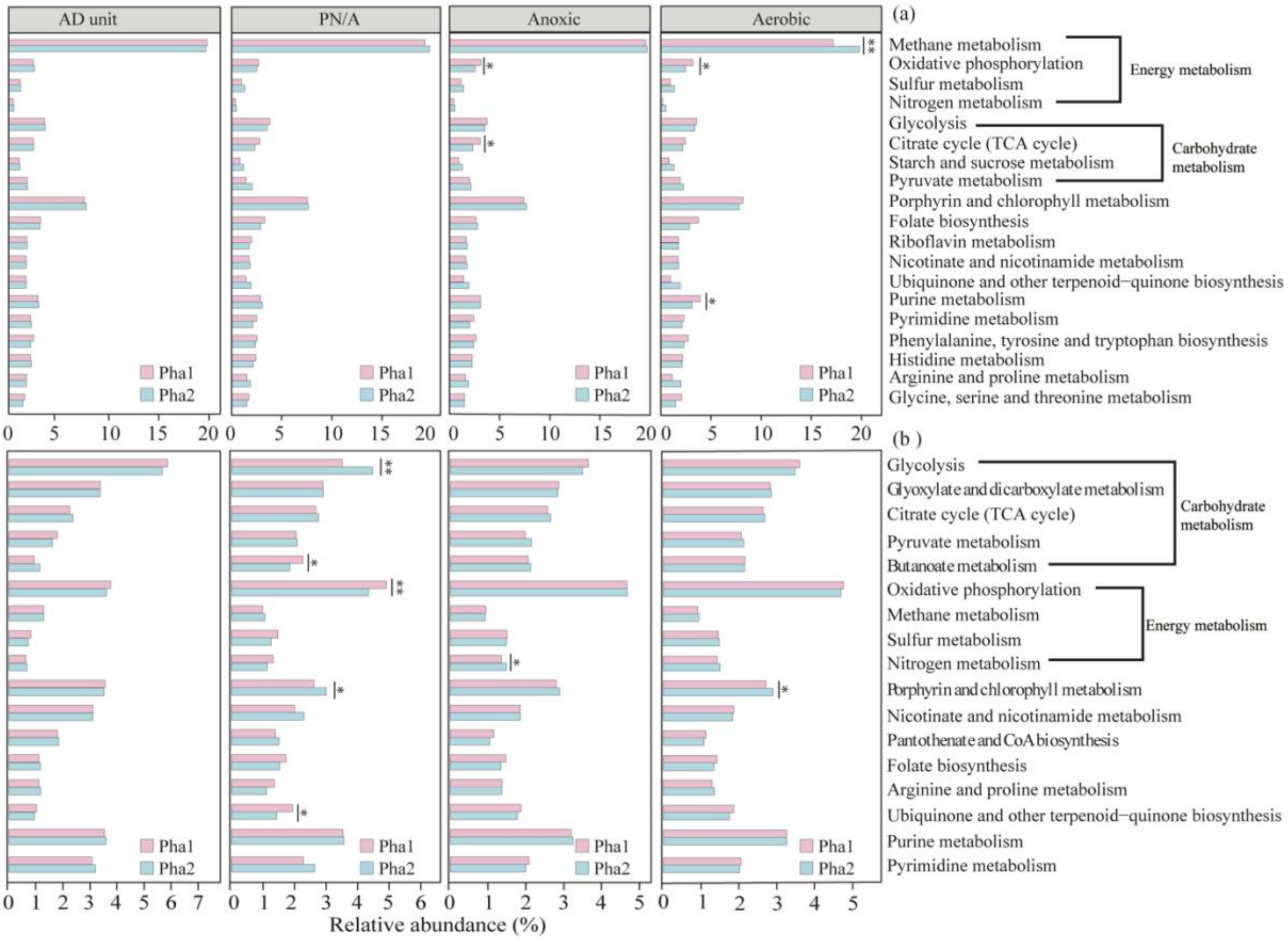

Fig. S5 Relative abundance of KEGG metabolic pathways at level3. (a) carried out by archaea. (b) carried out by bacteria. * $p < 0.05$; ** $p < 0.01$; *** $p < 0.001$. Statistical comparisons were based on n=3 independent samples per phase.

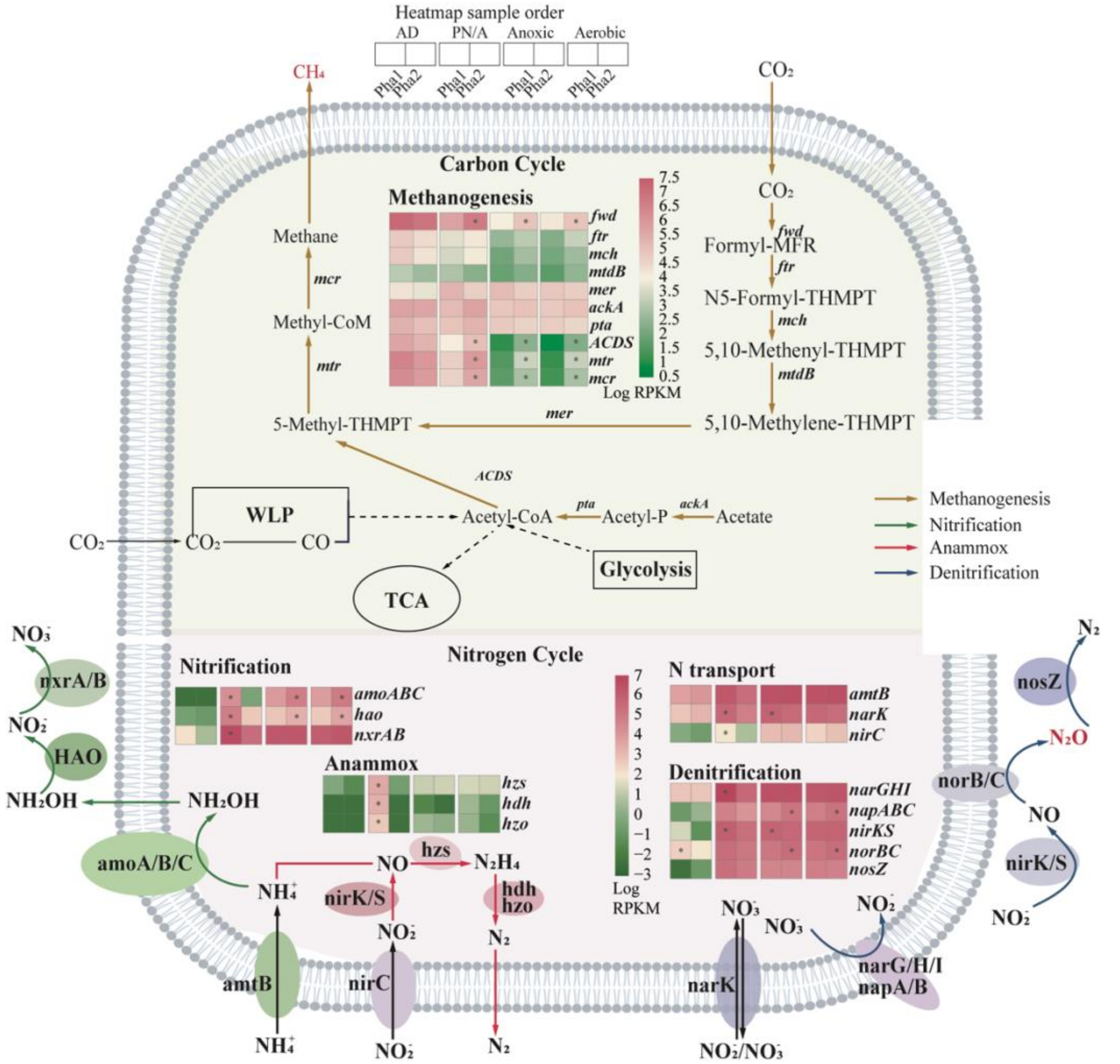

Fig. S6 CH4 and Nitrogen metabolic pathways and related functional gene abundances. Gene abundance refers to the overall abundance of a given gene across the entire microbial community, rather than within a specific microorganism. The colour key indicates the abundance calculated as PRKM in the log scale.

Roothans et al. reported that the ratio between *nirK/S* and *nosZ* in denitrifiers typically remains relatively stable (0.5 - 1.5) across seasonal changes,[4] indicating denitrifiers are unlikely to be the main N_2O producers. Ratios between the abundance of *nirK/S*(total) (AOB + NOB + denitrifiers), *nirK/S*(DEN) (denitrifiers only), and *nirK*(nitrifier) (AOB + NOB) and the only known N_2O-reducing enzyme *nosZ* were calculated (Fig. S7; Supplementary Data 4). The ratio of *nirK/S*(total) to *nosZ* increased from 5.9 and 3.7 to 7.5 and 5.0, while the ratio of *nirK/S*(DEN) to *nosZ* declined from 3.0 and 1.6 in Phase1 to 2.4 and 1.0. Meanwhile, the ratio of *nirK*(nitrifier) to *nosZ* increased markedly, from 3.0 and 2.2 in Phase1 to 5.1 and 4.0 in Phase2 in the Anoxic and Aerobic tanks, respectively. Furthermore, *nirK*(nitrifier) and *nirK/S*(DEN) increased from 1.0 and 1.6 in Phase1 to 2.1 and 4.1 in Phase2, suggesting a relatively greater representation of nitrifier-denitrification genes compared with denitrifier *nirK/S* in Phase2.

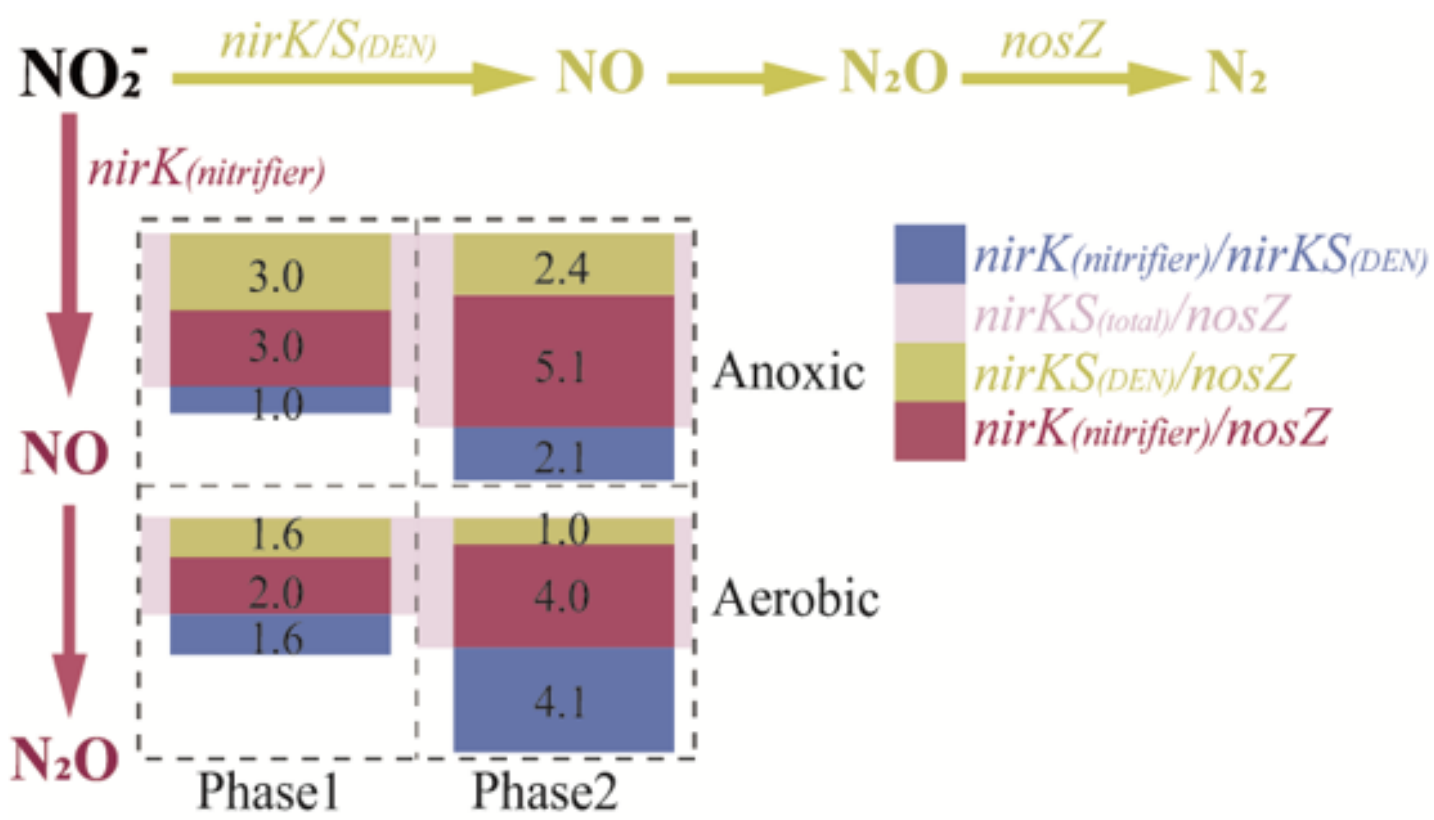

Fig. S7 Ratios between nitrite consuming enzyme *nirK/S* and N_2O reducing enzyme *nosZ* in the Anoxic and Aerobic tanks. *nirK*(nitrifiers) refers to *nirK* in AOB *Nitrosomonas* and NOB *Nitrospira*. *nirK/S*(DEN) refers to *nirK/S* in denitrifiers only. *nirK/S*(total) refers *nirK/S* in AOB, NOB, and denitrifiers. Values refer ratios between genes.

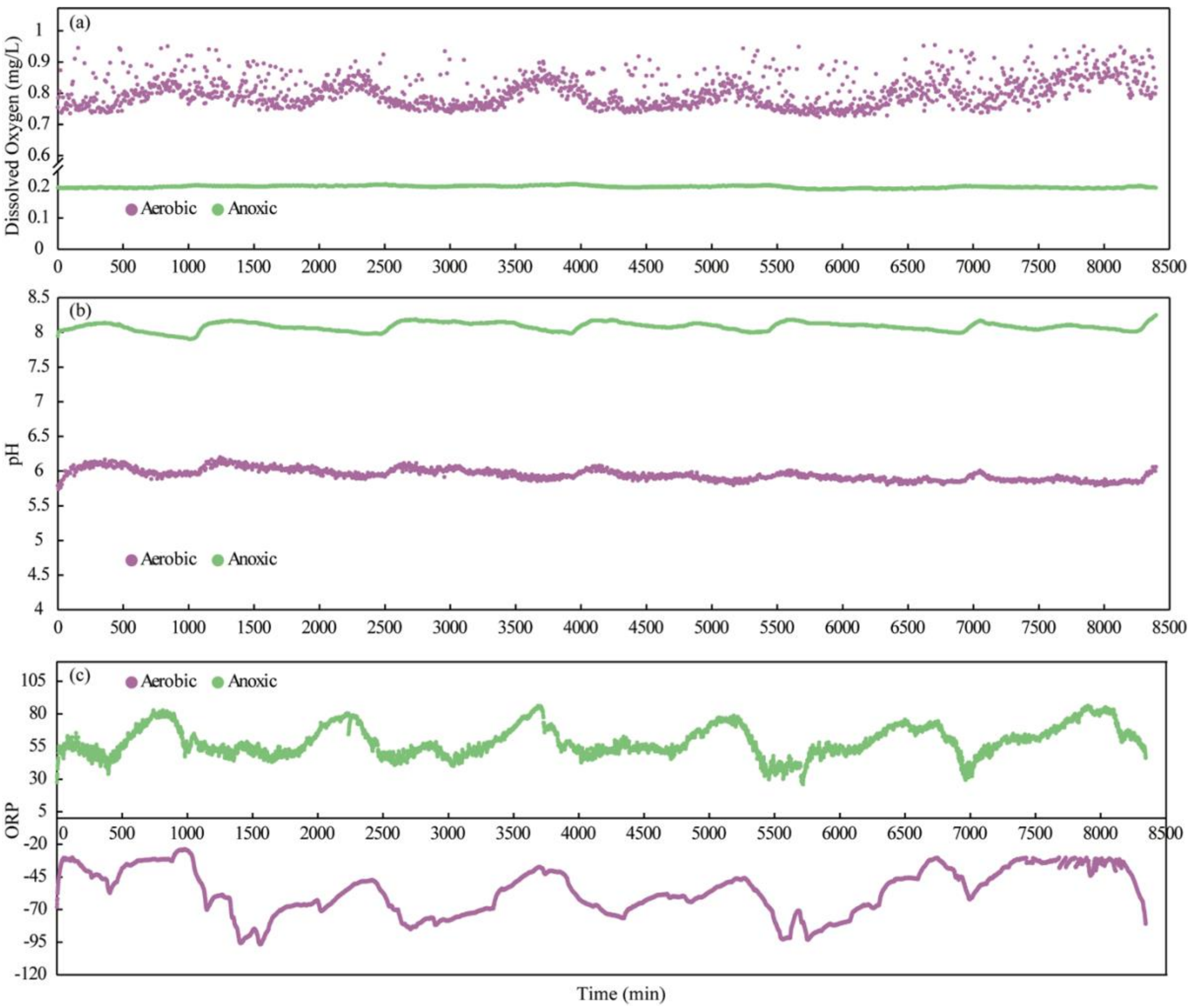

Fig. S8 Operational stability of dissolved oxygen, pH, and ORP in the Anoxic and Aerobic tanks during the study period. (a) Dissolved oxygen (DO). (b) pH. (c) Oxidation–reduction potential (ORP) monitored in the Anoxic and Aerobic tanks throughout operation. pH remained consistently higher in the Anoxic tank (green; ~7.9–8.2) than in the Aerobic tank (purple; ~5.7–6.1)**,** consistent with alkalinity recovery in the Anoxic tank and alkalinity consumption in the Aerobic tank.

Electricity generation from produced CH_4 increased from 26274.6 kWh in Phase1 to 34858.0 kWh in Phase2.

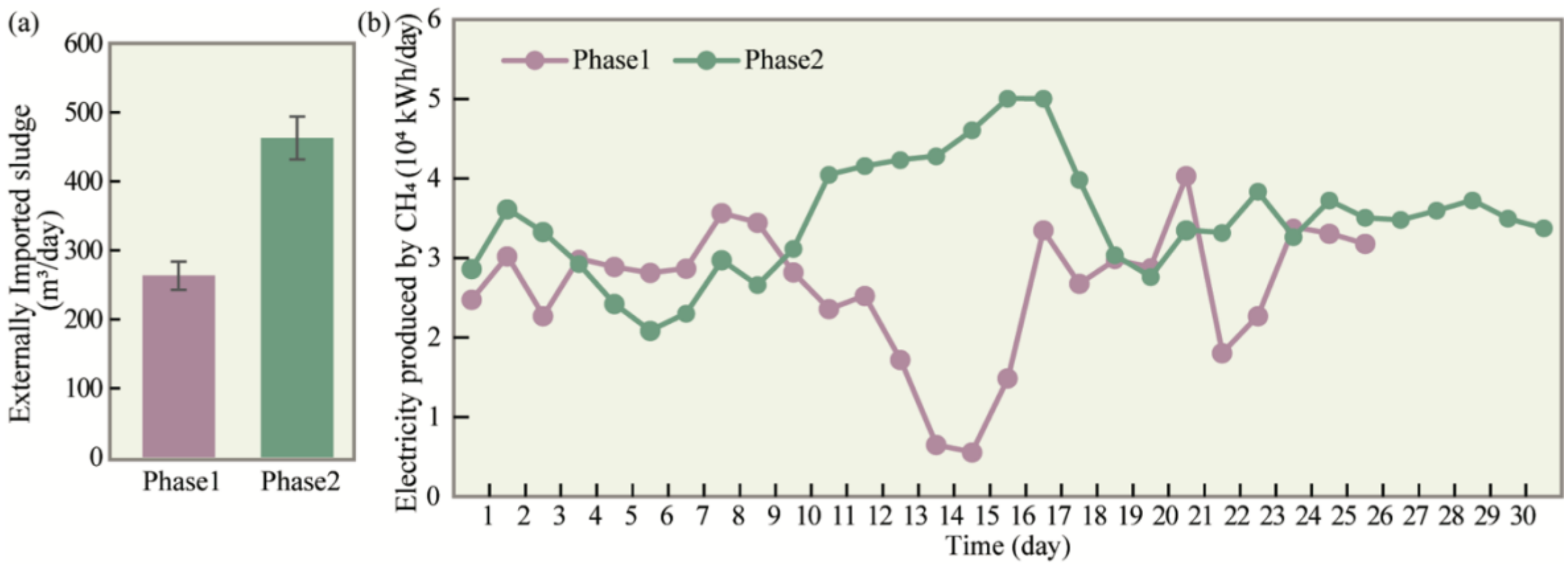

Fig. S9 Imported sludge loading and electricity generation from AD biogas-derived CH_4 during the two operational phases. (a) Volume of externally imported sludge for biogas recovery during the two sampling phases. (b) Monthly electricity generation from AD biogas-derived CH_4 during the two sampling phases. For each phase, 30 days of data are presented. Data averaged over one month were used for comparison between two phases.

Table S1. UK Government GHG Conversion Factors and GHG offset using electricity produced from CH_4 across two phases.

Activity	Country	CO_2-eq.kg	CO_2-eq.kg of CO_2 per unit	CO_2-eq.kg of CH_4 per unit	CO_2-eq.kg of N_2O per unit	Unit
Electricity generated	Electricity: UK	0.17700	0.17489	0.00090	0.00122	kWh
Biogas	Biogas: UK	0.00022	-	-	-	kWh
	Electricity generation from AD biogas-derived CH_4	CO_2-eq.kg produced – biogas	CO_2-eq.kg produced – electricity	Offset CO_2-eq.kg		
Phase1	26274.6 kWh	5.78	4650.6	1517.4		
Phase2	34858.0 kWh	7.67	6169.9			

Table S2. Operation parameters for N_2O emissions calculation in side-stream and mainstream.

	Process	N removal percentage by pathway	TN removed	Flow rate	N_2O produced	N_2O produced in total	N_2O emission rate
		(%)	(mg-N/L)	(m^3/day)	(g-N/($m^3 \cdot d$) N_2O)	(g-N/($m^3 \cdot d$) N_2O)	kg-N_2O/day
Phase1	PN/A	80.1	1810.5	650.0	5.84	9.59	6.23
	Denitrification	19.9			3.75		
Phase2	PN/A	42.6	742.0	668.1	1.31	5.86	3.81
	Denitrification	57.4			4.55		
Phase1	Mainstream	-	-	49727.0	-	-	-
Phase2	Mainstream	-	-	41132.1	-	-	-

Note: The percentage of nitrogen removal by each pathway in PN/A tank was predicted based on specific nitrogen removal activity results (Fig. S1).

Table S3. Alpha diversity indexes.

Alpha diversity indices, including Chao1, (richness), Pielou (evenness), Shannon (combined richness and evenness), and Simpson (dominance), were used to characterize microbial community structure in each tank across the two phases. In Phase1, Chao1 index was 132 in the AD unit and 295 in the PN/A tank, both slightly reduced to 126 and 290, respectively, in Phase2, indicating a reduction in microbial richness in these tanks. Conversely, Chao1 increased from 520 and 501 in Phase1 to 563 and 544 in Phase2 in the Anoxic and Aerobic tanks, respectively, suggesting enhanced microbial richness in PN/A downstream tanks. The Pielou and Shannon indices slightly increased in the AD unit (Pielou: 1.06-fold, Shannon: 1.12-fold) but slightly decreased in the PN/A, Anoxic, and Aerobic tanks in Phase2 (ranging from 1.9 – 6.0% compared to Phase1). Meanwhile, the Simpson index exhibited marginal changes, with a 1.00- to 1.03-fold increase in Phase2. Overall, reduced evenness and diversity in AD unite downstream three tanks implies that some specialist taxa may have been favored in Phase2, leading to dominance of specific microbial groups and a corresponding decline in overall diversity.[5]

		**AD unit**			**PN/A**			**Anoxic**			**Aerobic**		
**Chao1**	Phase1	S01	S02	S03	S04	S05	S06	S07	S08	S09	S10	S11	S12
		133	144	120	299	280	307	478	556	527	503	475	526
	Phase2	S201	S202	S203	S204	S205	S206	S207	S208	S209	S210	S211	S212
		126	132	122	273	256	343	544	605	541	541	508	582
**Simpson**	Phase1	S01	S02	S03	S04	S05	S06	S07	S08	S09	S10	S11	S12
		0.81	0.77	0.78	0.88	0.91	0.90	0.95	0.95	0.96	0.95	0.97	0.97
	Phase2	S201	S202	S203	S204	S205	S206	S207	S208	S209	S210	S211	S212
		0.81	0.73	0.81	0.87	0.91	0.87	0.93	0.94	0.92	0.93	0.94	0.95
**Pielou**	Phase1	S01	S02	S03	S04	S05	S06	S07	S08	S09	S10	S11	S12
		0.45	0.45	0.44	0.50	0.54	0.52	0.63	0.61	0.66	0.64	0.70	0.67
	Phase2	S201	S202	S203	S204	S205	S206	S207	S208	S209	S210	S211	S212
		0.50	0.43	0.49	0.50	0.56	0.47	0.63	0.62	0.60	0.62	0.64	0.63
**Shannon**	Phase1	S01	S02	S03	S04	S05	S06	S07	S08	S09	S10	S11	S12
		2.20	2.22	2.12	2.84	3.05	3.00	3.86	3.88	4.14	3.95	4.30	4.19
	Phase2	S201	S202	S203	S204	S205	S206	S207	S208	S209	S210	S211	S212
		2.41	2.10	2.35	2.82	3.11	2.77	3.95	3.97	3.79	3.92	3.97	3.98

Table S4. Genus-level assignment of methanogenesis-related genes in the AD unit.

	Methanothrix		*Methanobacterium*		*Methanoculleus*		*Methanosarcina*		Total	
	Pha1	Pha2	Pha1	Pha2	Pha1	Pha2	Pha1	Pha2	Pha1	Pha2
ackA	0.0	0.0	0.0	0.0	0.0	0.0	39.1	23.1	39.1	23.1
pta	0.0	0.0	0.0	0.0	0.0	0.0	39.6	23.7	39.6	23.7
cdh	0.3	0.8	17.3	11.5	4.0	2.9	199.1	120.5	275.9	169.4
fwd	0.0	0.4	143.8	89.8	29.2	28.1	785.3	481.8	1077.5	679.8
ftr	0.1	0.1	0.0	0.0	2.2	2.2	43.2	26.4	64.5	41.9
mch	0.0	0.0	19.5	11.6	1.9	1.9	41.7	24.0	63.1	37.6
mtd	0.0	0.0	0.0	0.0	0.4	0.3	41.8	26.0	64.3	40.7
mer	0.0	0.1	18.0	10.8	0.4	0.3	40.3	24.9	60.7	38.1
mtr	0.0	0.7	151.0	96.5	18.3	21.7	86.0	50.8	276.9	183.0
mcr	0.1	0.6	123.6	74.5	8.3	8.5	314.2	193.9	506.2	317.9

Note: Pha1 and Pha2 refer to Phase1 and Phase2, respectively. Gene abundance is calculated as RPKM. Total represents total abundance of the gene in the community.

Table S5 Size and operational parameters of each tank.

	PN/A	Anoxic	Aerobic
Depth, $\mathbf{D_R}$ (m)	3	3.5	3.5
Surface area (m^2)	300	530	5800
Volume, $\mathbf{V_R}$ (m^3)	900	1855	20300
pH	-	7.9~8.2	5.7~6.1
DO	-	0.19~0.21	0.72 ~ 1.02
ORP	-	-96.6~-23.4	26.2 ~ 86.5

Note: “-” represents that parameter was not routinely recorded onsite in the specific tank.

Table S6 Phase classification

	Phase1 (Satisfied PN/A)	Phase2 (Disturbed PN/A)
Period	Mar-2023	Aug-2023
TN of crude sewage (mg-N/L)	43.0	34.5
COD of crude sewage (mg/L)	524.2	549.0
Mainstream influent flow (m^3/day)	41439	34277
TN of mainstream influent (mg-N/L)	37.3	38.9
PN/A influent flow (m^3/day)	650.0	668.1
PN/A NH_4^+ (mg-N/L)	256.7	577.1
NH_4^+ volumetric loading ($kg\text{-}N \cdot m^{-3} \cdot d^{-1}$)	1.5	1.0
NH_4^+ removal rate ($kg\text{-}N \cdot m^{-3} \cdot d^{-1}$)	1.3	0.5
AD OLR ($kg\ VS \cdot m^{-3} \cdot d^{-1}$)	~2.15	~3.35

Table S7. Nitrogen and COD dosing for specific activity tests.

	Phase	Anammox	Denitrification	Nitrification
NH_4^+-N (mg/L)	Phase1	120	-	240
	Phase2	240		480
NO_2^--N (mg/L)	Phase1	160	160	-
	Phase2	300	300	
COD (mg/L)	Phase1	-	300	-
	Phase2		500	

Table S8. Sludge sample information for DNA extraction.

Sampling Phase	**Tank**			
	AD unit	PN/A	Anoxic	Aerobic
Phase1	S01, S02, S03	S04, S05, S06	S07, S08, S09	S10, S11, S12
Phase2	S201, S202, S203	S204, S205, S206	S207, S208, S209	S210, S211, S212

Table S9. On site information and constant value calculation for greenhouse gases emission calculation.

**Onsite Parameters**				**Calculation Parameters**		**Constant Value**	
	PN/A	Anoxic	Aerobic				
Depth, $\mathbf{D_R}$ (m)	3	3.5	3.5	$\boldsymbol{K_L\alpha_{N_2O,20℃}}$ (/d)	81.87	$\mathbf{C_{N2O,\ air}}$ (g-N/m^3)	0.0003
Surface area (m^2)	300	530	5800				
Volume, $\mathbf{V_R}$ (m^3)	900	1855	20300	$\boldsymbol{K_L\alpha_{N_2O,process}}$ (/d)	69.35	$K_L\alpha^{non-aerated}_{N_2O,T_{Process}}$ (/d)	2
Temp, $\mathbf{T_{Process}}$ (°C)	33.0	13.0	13.0				
Total daily airflow, $\mathbf{Q_A}$ (m^3/day)	1020000			$\boldsymbol{K_L\alpha_{O_2}}$ (/h)	0.16	$\mathbf{H_{N2O}}$	0.025
Superficial gas velocity, $\mathbf{v_g}$ (m/s)	0.002035441						
Input power, **P** (W/m^3)	8.0			$\boldsymbol{K_L\alpha_{CH_4}}$ (/h)	0.14		

Supplementary Data file (Supplementary Data.xlsx) description

Supplementary Data 1: Functional organisms' composition at genus level (relative abundance %).

Supplementary Data 2: Abundance of nitrogen cycle related functional genes (RPKM).

Supplementary Data 3: Abundance of N_2O emission related functional genes in specific bacteria in the Anoxic and Aerobic tanks.

Supplementary Data 4: Ratios between nitrite consuming enzyme *nirK/S* and N_2O consuming enzyme *nosZ.*

References:

(1) Liu, Z., et al. Formation characteristics of algal-bacteria granular sludge under low-light environment: From sludge characteristics, extracellular polymeric substances to microbial community. *Bioresource. Technol.* **376**, 128851 (2023).

(2) Zhang, K., et al. Genome-centered metagenomics analysis reveals the microbial interactions of a syntrophic consortium during methane generation in a decentralized wastewater treatment system. *Appl. Sci-Basel.* **10**, 135 (2020).

(3) Shi, K., et al. Regulating microbial redox reactions towards enhanced removal of refractory organic nitrogen from wastewater. *Water Res.* **258**, 121778 (2024).

(4) Roothans, N., et al. Long-term multi-meta-omics resolves the ecophysiological controls of seasonal N_2O emissions during wastewater treatment. *Nat. Water* **3**, 590-604 (2025).

(5) Gao, M.J., et al. Deciphering the role of granular activated carbon (GAC) in anammox: Effects on microbial succession and communication. *Water Res.* **233**, 119753 (2023).
